# How do employees think the COVID-19 crisis will affect their careers?

**DOI:** 10.1371/journal.pone.0246899

**Published:** 2021-05-06

**Authors:** Louis Lippens, Eline Moens, Philippe Sterkens, Johannes Weytjens, Stijn Baert

**Affiliations:** 1 Department of Economics, Ghent University, Ghent, Belgium; 2 Department of Sociology, University of Antwerp, Antwerp, Belgium; 3 Department of Economics, Université catholique de Louvain, Louvain-la-neuve, Belgium; 4 IZA Institute of Labour Economics, Bonn, Germany; 5 Global Labour Organization, Maastricht, The Netherlands; 6 IMISCOE, Rotterdam, The Netherlands; TED University, TURKEY

## Abstract

This study is the first in the world to investigate the expected impact of the COVID-19 crisis on career outcomes and career aspirations. To this end, high-quality survey research with a relevant sample of Flemish (Belgian) employees was conducted. About 21% of them fear losing their jobs due to the crisis—14% are concerned that they will even lose their jobs in the near future. In addition, 26% expect to miss out on promotions that they would have received had the COVID-19 crisis not occurred. This fear of a negative impact is higher in vulnerable groups, such migrants. In addition, we observe that many respondents believe they will look at the labour market differently and will have different work-related priorities in the future. In this respect, more than half of the respondents indicate that they have attached more importance to working conditions and work-life balance since the COVID-19 crisis.

## Introduction

Respected international organisations agree that the current COVID-19 crisis will have a tremendous impact on society at large, both in the short and in the long [1–3]. In March and April of 2020, hospitals in several countries have been operating at their maximum capacity, and thousands of deaths have occurred. These countries have also gone into lockdown to varying degrees, which means that people have to live in social isolation from each other [2–4]. Based on systems of ‘temporary unemployment’, employment contracts have been suspended in companies that are unable to comply with social distancing directives, or which have experienced an immediate fall in demand for their products and services [1, 5]. In the longer term, there is a fear that what began as a health crisis will develop into a deep economic crisis, with a decline in growth in the long term and increasing unemployment [1, 4–6].

With regard to the specific situation in Belgium, as of the 14th of April 2020, at the time of writing, the country had 31,119 confirmed cases of COVID-19 [7]. A slight majority (59%) of the infected people lived in Flanders. Therefore, approximately 0.27% of the population was infected, which made Belgium the fifth worst affected country in terms of infections per capita [8]. Since the 11th of March 2020, basically only hospitalised patients and medical personnel have been tested for COVID-19 [9]. Accordingly, the reported numbers should be considered as lower bounds and not as true values. Belgium has been in lockdown since the 18th of March 2020. Citizens are only allowed to leave their residences to purchase essential groceries. Working is allowed, but all firms are asked to allow most of their employees to work from home. When this is not possible, only firms that respect the social distancing rules can allow their employees to continue working. Despite the policy to allow people to continue working in Belgium, COVID-19 is causing a tremendous increase in unemployment. The Belgian system of temporary unemployment ensures a replacement income for employees without the risk of these employees to be laid off. While no detailed statistics were available at the time of writing, at least 1,258,000 people have been confirmed as registering as ‘temporarily unemployed’ in Belgium by the 1st of April 2020 (source: Eurostat and General Directorate of Statistics—Statistics Belgium) which, compared to the size of the labour force, is a substantially higher figure than are the comparable numbers in France and Germany [2]. The sharp increase in the number of unemployed people relative to neighbouring countries is most likely due to a number of reasons: (i) The national employment office has simplified the procedure for requesting unemployment benefits to a significant degree, ruling out the need for firms to fill in monthly control forms in order to qualify for the unemployment benefits, (ii) there are significant cumulative benefits attached to the statute of temporary unemployment as the benefits have increased with 5%. (from 65% to 70% of the limited gross salary) due to the COVID-19 crisis, (iii) in addition, the temporary unemployed can exceptionally enjoy a small daily premium of EUR 5.63 (per workday) and a reduction in the costs of social rent and utilities.

However, the quantitative predictions about the actual (long-term) impact of the COVID-19 crisis on the economy and labour market in the OECD countries, including Belgium, vary dramatically. This is not surprising. Since standard economic models are trained using data from ‘normal times’, making predictions concerning ‘abnormal times’ is challenging for economic research. Moreover, the extent to which the supply side of the labour market will react to this health shock is unclear, particularly whether employees will adjust their labour preferences and corresponding behaviours.

To the best of our knowledge, the present research is the first study in the world to investigate the expected impact of the COVID-19 crisis on career outcomes and career aspirations at the regional level (i.e. Flanders, Belgium) using standardised measurements. Specifically, survey responses from a post-stratified sample of 3,821 employees, representative by gender, age and education of the broader Flemish population of employees, provide answers to two research questions.

Research question 1 (RQ1): What do Flemish employees think the impact of the COVID-19 crisis will be on their careers?

Research question 2 (RQ2): What do Flemish employees think the impact of the COVID-19 crisis will be on the importance they attach to various job characteristics?

The answers to these research questions are indispensable to both firms and governments. First, we explore the existence of several career-wise fears possibly due to the COVID-19 crisis (amongst others the fear of job loss and the fear of missing out on promotions). Detecting these fears is important because perceptions of job insecurity are known to negatively impact ‘organizational’ outcomes (e.g. job satisfaction) and ‘extraorganizational’ outcomes (e.g. employee well-being), economic behaviour (e.g. spending and saving decisions) and major life decisions (e.g. getting married and buying a house) [10–13]. The early detection of perceptions related to the fear of job loss amongst employees can prevent an overall climate of job insecurity because these perceptions are known to reinforce the detrimental effects of job insecurity [10]. Moreover, employee expectations in general encompass substantial predictive power about the future. Dées and Soares Brinca [14], for example, state that confidence indicators–which are also mainly based on expectations–are thought to predict periods of strong fluctuations in the economy, such as recessions and recoveries. Also, Hurd and Rohwedder [12] asked the American Life Panel, in the beginning of the global financial crisis of 2008, on their subjective probability of becoming unemployed in the next 12 months. The authors state that workers predicted quite accurately on average what their unemployment experience would be. In general, recessions have a major impact on the decisions people make in their careers [15, 16]. Therefore, a government that is aware of said career-wise fears (e.g. the fear of job loss) in general and amongst specific subgroups (e.g. at the sector level) at the start of a crisis can take this into account in its economic support program. Second, to counter the negative consequences such as lower employee well-being or job satisfaction, it is important to know if the COVID-19 crisis has changed priorities when it comes to valuing different aspects of a job. This, too, can be of significant importance for the government’s economic revival strategy. More generally, the answers to our research questions enables the government to discover if action is necessary, for which subgroups specifically and which course should be set to undertake successful and targeted actions.

## Data

### Research population and sampling

The research was conducted in Flanders, the largest of three Belgian regions. Our research population consisted of Flemish employees under the age of 65, which is the legal retirement age in Belgium. This delineation is in line with our research focus on employees (versus the self-employed) and allowed us to compare the sample’s characteristics with population averages, as discussed below. Furthermore, the research was conducted in a faculty of economics and business administration outside the United States, where ex ante ethical approval of survey research with informed consent and without sensitive topics or the aim of merging with other data is not asked for.

The labour market in Flanders is characterised by two main features. First, the competition for human capital is relatively high in comparison to other regions in Europe [17, 18]. More specifically, this high level of competition is indicated by the combination of two parameters: the job vacancy rate and the employment rate. In the fourth quarter of 2019, the job vacancy rate—that is, the number of vacancies as a percentage of the sum of this number and the number of occupied jobs—was 3.9% in Flanders, while it was 2.2% in the EU-27 (source: General Directorate of Statistics—Statistics Belgium). Next, the overall employment rate of the Flemish population aged 20 to 64 was 75.5% in the fourth quarter of 2019, while it was 70.5% in the EU-27 (source: Eurostat and General Directorate of Statistics—Statistics Belgium).

Second, in Flanders, labour contracts are highly regulated [17]. Nearly every employer (approximately 96%) is bound by a collective labour agreement negotiated by the labour unions [19]. The negotiations leading up to these agreements are held by joint committees (under which both employer and union representatives reside) at several levels (at the national, sector and firm levels) [20]. Eventually, these negotiations result in agreements related to wage and labour conditions to which every employer has to adhere in the sectors or firms covered by the respective committees [20]. These two distinctive features of the Flemish labour markets, high competition for human capital and high labour market regulation, are indicative of our findings’ external validity because one could expect more similar findings in labour markets that are comparable to that of Flanders.

Under ideal research circumstances, the ‘first-best’ option to arrive at a representative sample of our population would have been to draw a probability sample from all Flemish employees under the age of 65. If we had been able to draw such a—sufficiently large—sample and have the participants answer our survey effectively—this second condition is often not met in studies that label their data as representative—we would be confident that all the reported findings would be perfectly representative of our population [21–23]. However, this sampling procedure is only possible at a high cost and within a long-term research project (for example, through sampling via the national register, after ethical approval, and the follow-up by the register office of non-responses of the participants), which was neither feasible nor desirable in our case because we wanted to inform the scientific community and policy makers about career expectations at the micro level as quickly as possible.

Our research was based on a web survey. Participants could access the web survey via a weblink that would lead them to the Qualtrics website. This weblink was featured in a newspaper article, published via the largest newspaper outlet in Flanders, announcing the survey (title translated from Dutch: “What will happen with your career following the corona crisis? Ghent University wants your help in researching the impact of the corona virus on the labour market.”). In the announcement, participants were briefly informed about the study’s goal and its target population. According to the latest figures of the Belgian centre for media information (‘Centrum voor Informatie over de Media’, CIM) the particular media outlet has a coverage of a quarter of the (online and offline, primarily Flemish-speaking) newspaper readers in Belgium with no substantial under-representation across age groups, social groups, (Flemish) provinces, professions or educational attainment (for the complete overview of readership statistics we refer to the CIM figures) [24]. Featured on the newspaper’s website and social media, the article was released open access and was therefore accessible to the general public (both subscribers and non-subscribed readers). The newspaper’s contribution to this study was strictly limited to the distribution of the study via their various online channels and editing the announcement in their article. Its incentive for contributing to the study was the right to be the first outlet to publish the results from the survey in a news article.

This method of data gathering has several advantages. For example, as respondents are able to determine the timing and pace of web surveys themselves, and because they can reread questions easily, they will give more reliable and valid answers compared to when engaging in physical or telephonic interviews [21, 23]. However, there are also two important drawbacks with the use of web surveys [21, 23]. The first is the problem of the under-representation of groups that do not have access to the Internet (‘undercoverage’). However, calculations based on the European Social Survey (round 9 of 2018) show that the distortion caused by this problem is negligible, as the number of Belgian employees under the age of 65 who never use the Internet is only 1%—in addition, 96% indicate that they use the Internet several times per week and 3% indicate to do so less frequently. In order to address this issue further, in our calls for the survey, which were supported by the largest Belgian newspaper, we repeatedly underlined that we hoped that readers of our call would not only complete the survey themselves, but would also encourage their less digital-savvy acquaintances to do so.

A more important problem that compromises the overall representativeness of our sample—and that of almost all web surveys—is linked to the fact that the respondents themselves chose whether or not to participate in our survey (‘self-selection’). The answers of those who actually participated might be different from the responses of those who did not choose to complete the survey. Therefore, to enhance the representativeness of our survey, we followed the strategy of post-stratification, as recommended by Bethlehem and Biffignandi [21] and Tourangeau and colleagues [23], two seminal works on web surveys. Under certain conditions (see below), this strategy completely eliminates any bias caused by self-selection (and under-representation); in practice, empirical work has found that, on average, the strategy leads to significant reductions in bias [23].

In our case, post-stratification entailed the selection of a sub-sample of 3,821 individuals resembling the population of Flemish employees under 65 years of age in terms of (i) gender, (ii) age, and (iii) education level from our total sample of 14,005 respondents. These three ‘auxiliary variables’ were hypothesised—and found, as discussed below—to have great predictive power with regard to the constructs we wished to investigate in the context of answering RQ1 and RQ2. Specifically, we aimed for representativeness by two levels for each of these three variables: males versus females, tertiary education versus non-tertiary education and being younger or older than 50. We were bound by these specific levels (per grouping variable) due to the availability restrictions of Flemish population data by stratum, which were provided to us by the General Directorate of Statistics—Statistics Belgium (see S1 Table O in S1 File for a brief overview of these statistics). By combining these levels, we created eight—two times two times two—cells (‘strata’) amongst which we wanted to realise a balance. Including more strata was found to be inappropriate because it resulted in an insufficient number of individuals per stratum [21, 23]. Next, we identified the stratum that was most underrepresented in comparison to population averages for all Flemish employees under 65 years of age in 2019. This was revealed to be the stratum of female workers without a tertiary education diploma and aged 50 or older. All the individuals with complete answers who met our inclusion criteria were included in the final subsample for this stratum. For the other seven strata, individuals were selected randomly according to their proportions in the population (compared to females without a tertiary education diploma and aged 50 or older). Accordingly, our post-stratified sample, like the wider population, consists of:

(i) 22.2% (13.7%) males without (with) tertiary education qualifications being younger than 50;
(ii) 9.8% (5.4%) males without (with) tertiary education qualifications being older than 50;
(iii) 15.6% (19.3%) females without (with) tertiary education qualifications being younger than 50; and
(iv) 8.3% (5.7%) females without (with) tertiary education qualifications being older than 50.

The results presented below are robust against analysing alternative random selections for the seven strata with an abundant supply in the full sample.

The assumption under which this post-stratification resolves the potential self-selection bias is that, within the strata that were formed, there is no longer a link between the chance of participating in the survey (selecting oneself to participate) on one hand, and the answers given for the central outcome variables of the survey on the other (the ‘missing at random’ assumption) [21, 23, 25]. An indication of the extent to which the ‘missing at random’ assumption is acceptable is the strength of the relationship between the auxiliary variables used for the post-stratification and the outcome variables of the survey [21]. A regression analysis showed that the characteristics across which we stratified did indeed have strong predictive power for many of the surveyed variables related to RQ1 and RQ2. For example, as can be seen in S1 Table A in S1 File, gender is a strong predictor of the fear of losing one’s job due to the COVID-19 crisis (*p* < 0.001 if no further control variables are included; *p* = 0.001 if controlling for all other personal and job characteristics adopted in the questionnaire). S1 Table P in S1 File provides the full correlation matrix for the variables ‘temporary unemployment’, ‘gender’, ‘age’ and ‘level of education’.

The results of the regression analyses presented below can be seen as an additional effort to address the potential self-selection bias, as these analyses indicate how our outcome variables evolve for different values of the auxiliary variables (‘generalised regression estimation’) [21, 23].

### Survey

The on-line survey was available for completion via Qualtrics between Wednesday 25 March 2020 and Tuesday 31 March 2020 via Qualtrics. The survey consisted of seven parts. First, two introduction screens were displayed. On the first introduction screen,

(i) the objective of the survey was explained (investigating the expected impact of the COVID-19 crisis on the careers of salaried employees);
(ii) the survey population was clarified; and
(iii) the incentives for completing the questionnaire were underlined (that is, committing oneself to important social research and having a chance of winning one of the 16 prizes to be raffled, with a combined value of EUR 688.40).

On the second introduction screen, respondents were informed about anonymised data processing and their rights as research participants in accordance with the GDPR guidelines at Ghent University, and were asked to agree to these modalities, thus providing their ‘informed consent’.

Second, the respondents were asked about their current work situation (employee, temporarily unemployed [due to the COVID-19 crisis], self-employed, classically unemployed, or inactive). Only those who fell into one of the first two categories could participate in the survey, in accordance with the call for this research.

In the third part, the respondents were questioned about the expected effects of the COVID-19 crisis for their careers. Specifically, the following seven potential fears were evaluated:

(i) losing job in the short or long term;
(ii) losing job in the short term;
(iii) missing out on promotion;
(iv) overall negative impact on career;
(v) negative impact on wage;
(vi) negative impact on personal motivation; and
(vii) negative impact on the number of attractive vacancies.

In the fourth part, the respondents were questioned about the expected impact of this crisis on the extent to which they attached importance to a series of job characteristics. Specifically, they were asked to share whether they would attach more or less importance to the following seven aspects of a possible new job as a result of the COVID-19 crisis:

(i) wage;
(ii) employment relationship;
(iii) job content;
(iv) working conditions;
(v) work-life balance;
(vi) distance to the workplace; and
(vii) possibility of teleworking.

Given the central position of the two latter parts of the survey within the context of the present article—these two parts delineate the outcome variables related to RQ1 and RQ2—the items adopted are included in S1 File.

Then, in the fifth part, the respondents were asked about their experiences, expectations and hopes with regard to (extended) telework (in the context of the COVID-19 crisis). These items have not been evaluated in the context of the present study, except for (i) the candidates’ assessments of the percentage of their work that could potentially be done via telework and (ii) an indicator of whether they had had to telework to an extended degree due to the COVID-19 crisis at the time of the survey. These variables were included in our analyses as potential moderators of expected career consequences (RQ1) and perceived evolutions in job priorities (RQ2).

In the sixth part of the survey, we gathered information about the personal characteristics of our respondents and the characteristics of their jobs in order to also investigate heterogeneity in the answers to RQ1 and RQ2 by these characteristics. Specifically, we gathered information concerning the respondents’ gender, age, migration background, education level, relationship status, number of resident children (and other persons), province and degree of urbanisation of their residence, and health status (prior to the COVID-19 crisis, overall current status and status as a COVID-19 patient), as well as their type of employment contract, the part-time nature of this contract, their tenure (with the current employer and in the current job), their level of job satisfaction experienced, four key characteristics of their job (autonomy, dependency on others, interaction outside of the organisation and feedback from others), and their sector of employment. The scales used (and their sources) are clarified in Table 1.

**Table 1:** Summary statistics (N = 3,821).

Female	0.488 (–)
Age	41.682 (10.851)
Migration background	0.027 (–)
Tertiary education	0.441 (–)
Single	0.202 (–)
In a relationship but not cohabiting	0.072 (–)
In a relationship and cohabiting	0.726 (–)
Number of resident children	0.911 (1.047)
Resident parents	0.077 (–)
Resident family members (other than parents)	0.038 (–)
Resident others (not family)	0.020 (–)
Province of Antwerp	0.279 (–)
Province of West Flanders	0.187 (–)
Province of East Flanders	0.307 (–)
Province of Limburg	0.075 (–)
Province of Flemish Brabant	0.152 (–)
Living in the countryside or rural area	0.358 (–)
Living in the centre of a village	0.272 (–)
Living in the suburbs of a city	0.225 (–)
Living in the centre of a city	0.146 (–)
Health before the COVID-19 crisis (scale)	4.106 (0.763)
Current health (scale)	3.910 (0.841)
Never been a COVID-19 patient (definitely or likely)	0.708 (–)
Uncertain about having been a COVID-19 patient	0.222 (–)
COVID-19 patient at the moment (definitely or likely)	0.037 (–)
COVID-19 patient in the recent past (definitely or likely)	0.033 (–)
Employed via a temporary contract in the private sector	0.041 (–)
Employed via a permanent contract in the private sector	0.787 (–)
Employed via a regular contract in the public sector	0.078 (–)
Employed via a permanent appointment in the public sector	0.093 (–)
Part-time contract	0.181 (–)
Tenure with current employer (scale)	2.915 (1.444)
Tenure in current job (scale)	2.471 (1.336)
Satisfied with job (scale)	3.992 (0.930)
Autonomous in job (scale)	3.774 (1.189)
Dependent on others in job (scale)	3.155 (1.107)
Interaction outside of the organisation in job (scale)	3.567 (1.393)
Feedback from others in job (scale)	3.065 (1.168)
Sector: Purchasing	0.010 (–)
Sector: Administration	0.071 (–)
Sector: Construction	0.033 (–)
Sector: Communication	0.016 (–)
Sector: Creative	0.008 (–)
Sector: Provision of services	0.076 (–)
Sector: Financial	0.050 (–)
Sector: Health	0.055 (–)
Sector: Catering and tourism	0.035 (–)
Sector: Human Resources	0.043 (–)
Sector: ICT	0.072 (–)
Sector: Legal	0.014 (–)
Sector: Agriculture and horticulture	0.003 (–)
Sector: Logistics and transport	0.074 (–)
Sector: Management	0.040 (–)
Sector: Marketing	0.017 (–)
Sector: Maintenance	0.018 (–)
Sector: Education	0.038 (–)
Sector: Research and development	0.025 (–)
Sector: Government	0.047 (–)
Sector: Production	0.069 (–)
Sector: Technology	0.040 (–)
Sector: Sales	0.099 (–)
Sector: Other	0.048 (–)
Temporarily unemployed	0.197 (–)
% of work potentially done via telework	42.795 (36.579)
Temporarily extended telework	0.503 (–)

Notes. No standard deviations are presented for binary variables. The levels (and values) for the health scales are: very bad (1), somewhat bad (2), neither bad nor good (3), somewhat good (4), and very good (5). The levels for the tenure scales are: less than 2 years (1), between 2 and 5 years (2), between 6 and 10 years (3), between 11 and 20 years (4), and more than 20 years (5). The levels for the job scales are: completely disagree (1), somewhat disagree (2), neutral (3), somewhat agree (4), and completely agree (5). The operationalisation of these variables is based on Amez, Vujić, Soffers, and Baert [31], Baert, Verhaest, Vermeir, and Omey [32], Baert and colleagues [33], Moens, Baert, Verhofstadt, and Van Ootegem [34], and Morgeson and Humphrey [35]. The summary statistics for age, gender and education are comparable to those in the general population by design due to the post-stratification. In 2018, 19.6% of all working Flemish citizens between 15 and 64 years of age had a migration background. The geographical spread of all working Flemish citizens between the age of 15 and 64, following the same order as the table, is 27.6%; 18.2%; 2.5%; 13.1% and 17.5%, respectively. Of these, 90.6% had a permanent contract and 9.4% a temporary contract, not taking into account the distinction between the private and public sector (Vlaamse Arbeidsrekening based on RSZ, RSVZ, RIZIV, RVA, Algemene Directie Statistiek–Statistics Belgium; https://www.steunpuntwerk.be/node/3588, https://statbel.fgov.be/nl/themas/werk-opleiding/arbeidsmarkt/werkgelegenheid-en-werkloosheid/plus). Similarly, in the first quarter of 2020 36.8% of working Flemish citizens enjoyed tertiary education. (Eurostat; https://appsso.eurostat.ec.europa.eu/nui/show.do?dataset=lfsq_pgaed&lang=en) Finally, in March of 2020, 19.6% of all Flemish working people were temporarily unemployed due to COVID-19. (Rijkdienst voor Arbeidsvoorziening; https://www.rva.be/nl/documentatie/statistieken/tijdelijke-werkloosheid-wegens-coronavirus-covid-19/cijfers).

Finally, in the seventh part of the survey, participants were presented with final screens on which they (i) were thanked for their participation and (ii) could leave their email address in the context of participation in future research and/or the lottery of prizes linked to full participation (as mentioned above).

Several precautions were taken to ensure the reliability and validity of the measuring instrument. In general, the guidelines in Bethlehem and Biffignandi [21], Fowler [22], and Tourangeau and colleagues [23], which are seminal handbooks concerning the drafting of questionnaires (for off-line and on-line surveys), were taken into account as far as possible when designing the questionnaire. In the following paragraphs, we discuss some of the decisions taken in this regard.

To counteract the problem of ‘non-differentiation’, whereby respondents, usually out of fatigue, start to fill in anything, the number of items within the same cluster and screen was limited [21]. Moreover, all the items were formulated comprehensibly, did not contain double-barrelled constructions or negations, and complicated wording was avoided as far as possible [26, 27]. As related research has indicated that rewarding respondents who complete surveys in full leads to better quality data, raffle prizes were offered to the participants (as mentioned earlier) [23, 28]. In addition, to encourage them to complete the questionnaire in full, a progress indicator for the questions was added [21]. Certain important words were presented in bold text [23].

In addition, we followed the advice of Weijters, Cabooter, and Schillewaert [29] with regard to response scales for research amongst the general public by using five-point Likert scales for the different items whenever possible. These scales usually comprised the answer options ‘completely disagree’, ‘somewhat disagree’, ‘neutral’, ‘somewhat agree’, and ‘completely agree’. There is no agreement on the use of ‘somewhat’ in the scientific literature. An argument for including it is that omitting it can lead to confusion. ‘Disagree’ can then be seen as the end point of the scale. For example, a respondent may (simply) not agree with the statement; whether he or she would ‘completely disagree’ or ‘[just] disagree’ may not be substantially different for some respondents, but completely different for others. This confusion may result in less frequent selection of the extremes, which may lead to ‘restriction of range’ (that is, the limitation of the probability of selecting certain response options).

Since we preferred to not only present regression analyses but also histograms capturing all the response options and their frequencies (see the Results section), it was important, in accordance with the advice of Weijters and colleagues [29], to label all response options. Moreover, in line with the recommendation of Weijters and Baumgartner [30], positively formulated items and negatively formulated items were alternated, as were regular-phrased and reversed-phrased items. In this way, the problem of ‘acquiescence’, whereby some respondents feel a strong tendency to agree with statements because they stop reading the questions meticulously at a certain point, was partly overcome [21]. Finally, we deliberately did not include the option ‘I do not know’ to prevent respondents from selecting this option quickly to avoid having to think about their actual responses [21].

The measuring instrument was refined on the basis of pilot tests, in several rounds, amongst 55 respondents. All feedback was recorded. In the later rounds, the focus was on the following questions:

(i) ‘Was it clear what was expected in each question?’
(ii) ‘Were there words or phrases that were unclear or difficult to understand?’ and
(iii) ‘Were there any crucial questions we forgot to pose given the aims of the survey?’

A ‘trap question’ was added to the questionnaire to test the participants’ attentiveness. Inattentive participants were not included in the cleaned dataset (and were therefore not eligible to be included in the post-stratified sample on which the results in this article are based). In the sensitivity analyses, respondents with the 5% shortest survey duration (that is, less than 6 minutes and 9 seconds) were excluded from the post-stratified sample. These exclusions did not change the results noticeably. The median time the respondents took to complete the survey was 10 minutes and 27 seconds. This median duration indicates that the duration of the survey was sufficiently limited to prevent ‘satisficing’; that is, less attentive answering of items, usually due to fatigue [21].

In Table 1, we provide the reader with some summary statistics concerning the resulting data.

## Results

### Perceived career-related fears induced by the COVID-19 crisis

**Fig 1:**
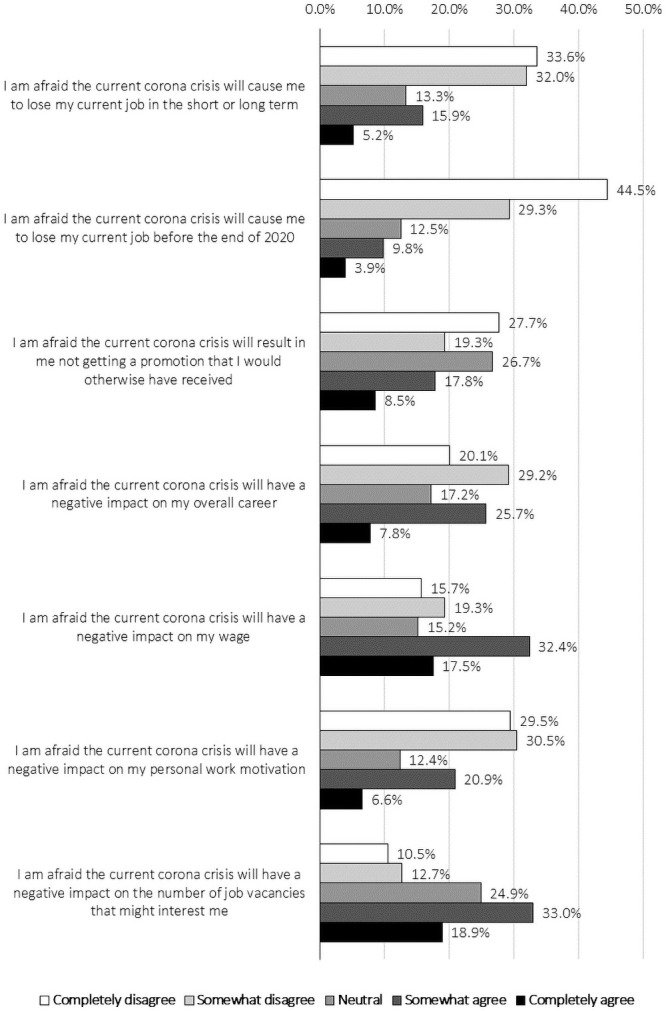
Perceived career-related fears induced by the COVID-19 crisis: Answers given.

Fig 1 provides an overview of the respondents’ responses to the items in the survey related to RQ1. Table 2 summarises the results of a regression analysis in which these responses were classified according to the personal and job characteristics surveyed. Linear regression analyses in which the standard errors were corrected for heteroscedasticity (White correction) were performed. Ordered logistic models and dummy specifications for the continuous explanatory variables included led to the same research insights. In addition, we computed multicollinearity diagnostics leading to variance inflation factors lower than 10 for each of the models presented. The complete regression results for the first item are included in S1 Table A in S1 File.

**Table 2:** Perceived career-related fears induced by the COVID-19 crisis: Regression results.

Fear	% with fear	Significantly more pronounced if …	Significantly less pronounced if …
Losing job in the short or long term	21.1%	Female; older age; migration background; province of Flemish Brabant; sector is catering and tourism; more dependent on others in job; temporarily unemployed.	Better current health; more secure contract (public sector or permanent contract in private sector); longer tenure with current employer; more satisfied with job; more feedback from others in job; sector is provision of services, financial, health, ICT, agriculture and horticulture or government.
Losing job in the short term	13.7%	Female; older age; province of Flemish Brabant; sector is catering and tourism or sales; temporarily unemployed; higher % of work potentially done via telework.	Better current health; more secure contract (public sector or permanent contract in private sector); longer tenure with current employer; more satisfied with job; more feedback from others in job; sector is provision of services, financial, health, ICT, agriculture and horticulture, maintenance, education or government.
Missing out on promotion	26.2%	Migration background; province of Flemish Brabant; more interaction outside of the organisation in job; sector is catering and tourism, human resources, ICT, legal, logistics and transport, production or technology; temporarily unemployed.	Older age; tertiary education; better current health; more secure contract (public sector or permanent contract in private sector); part-time contract; more satisfied with job; sector is health.
Overall negative impact on career	33.6%	Female; older age; province of West Flanders; province of Flemish Brabant; better health before the COVID-19 crisis; more dependent on others in job or more interaction outside of the organisation in job; sector is catering and tourism; temporarily unemployed.	Better current health; more secure contract (public sector or permanent contract in private sector); longer tenure with current employer; more satisfied with job; more feedback from others in job; sector is health or government.
Negative impact on wage	49.9%	Higher number of resident children; province of West Flanders; COVID-19 patient in the recent past (probably); more interaction outside of the organisation in job; sector is catering and tourism; temporarily unemployed.	In a relationship and cohabiting; better current health; more secure contract (public sector or permanent contract in private sector); more satisfied with job; more feedback from others in job; sector is health or government.
Negative impact on personal motivation	27.5%	Living in the suburbs of a city; better health before the COVID-19 crisis; more dependent on others in job.	Older age; better current health; more satisfied with job.
Negative impact on the number of attractive vacancies	51.9%	Tertiary education; province of West Flanders; province of East Flanders; better health before the COVID-19 crisis; more dependent on others in job; sector is creative or marketing; temporarily unemployed.	Female; older age; better current health; more secure contract (public sector or permanent contract in private sector); more satisfied with job; more feedback from others in job; sector is health, ICT, agriculture and horticulture or maintenance.

Notes. The proportion ‘with fear’ corresponds to the sum of those who indicated ‘completely agree’ and ‘somewhat agree’ to the related survey item (see S1 Text in S1 File). The relationship to the personal and job characteristics was analysed by means of a linear regression analysis with heteroscedasticity-robust standard errors (in which all characteristics mentioned in Table 1 were included). The significance level was set as *p* < 0.05. N = 3,821.

As can be seen in Fig 1 and Table 2, more than one in five (21.1%) of the respondents indicate to be afraid of losing their job due to the COVID-19 crisis. About one out of seven (13.7%) fear that this will even be the case before the end of the year. Notably, women express a more pronounced fear of job loss (in both the short and long term) and a negative impact on their careers while men are relatively more worried about the number of attractive vacancies available to them. Moreover, this fear is significantly more apparent in vulnerable groups, such as older employees and migrants. These results are in line with (i) the Eurofound survey carried out in April 2020 where nearly 10% of Belgians fear they will lose their job in the next 3 months and (ii) scientific research that indicates that the employment of disadvantaged groups is more sensitive to the economic cycle, and that discrimination on the labour market is greater when labour market tightness within occupations is lower [36–38]. Based on the further analysis of the data, the fear of losing one’s job permanently increases to four out of ten amongst the temporarily unemployed. This is somewhat in line with the scientific literature on the scarring effects of unemployment [39, 40].

Moreover, about one in four (26.2%) respondents fear to missing out on a promotion he or she would have received had the COVID-19 crisis not occurred. This fear is significantly higher amongst migrants and those who are currently temporarily unemployed, and significantly lower amongst those working in the public sector. One in two (49.9%) also fear a direct, negative effect of the crisis on their wages. This fear is significantly higher amongst those who have temporary contracts in the private sector or are currently temporarily unemployed. More than one in four (27.5%) fear an impact on their personal work motivation. This estimated impact is significantly higher amongst, inter alia, younger people. Finally, when compared to demographics such as gender and age, analyses on the heterogeneity of effects yields less statistically significant differences for level of education. However, we do find that, compared to those without tertiary education, workers who completed a tertiary education express less fear to miss out on a promotion.

In addition, more than half (51.9%) fear that the COVID-19 crisis will have a negative impact on the number of vacancies that might interest them. This fear of fewer attractive vacancies is significantly higher amongst highly educated people. This may seem surprising at first sight, but it might simply indicate that people with lower levels of education already feared a decline in the number of attractive vacancies before the COVID-19 crisis struck, given the reports of fewer job opportunities available to them due to digitisation and automation [41–44]. Our results seem to be in line with research carried out by Aucejo, French, Araya and Zafar [45], who have surveyed US students on the negative effects of the COVID-19 crisis on their graduation, job opportunities and wage expectations. While almost one in six students were confronted with a delayed graduation, 40% of them had lost out on a job opportunity (in our sample, 51.9% fear that there will be less interesting vacancies) and 29% expected to earn less early on in their career (in our sample, 49.9% fear a negative impact on wage).

The results described above illustrate the general fear of negative career consequences due to the COVID-19 crisis well. Similarly, about one in three (33.6%) fear an overall negative impact on their careers. In this respect, two sectors hit particularly hard are tourism and catering. Tourism worldwide faces an unprecedented setback due to the current COVID-19 crisis, as the World Tourism Organisation expects global tourism to fall by 20% to 30% this year compared to last year (figures as of 24 March 2020) [46]. In addition, the Flemish federation of pub, restaurant and hotel owners (‘Horeca Vlaanderen’) foresees similar declines in tourism and catering [47]. This negative impact on tourism and catering businesses, and by extension employers, also translates to the workforce, for whom, according to our results, the fear of overall negative career consequences is significantly higher than it is for employees working in most other sectors.

As a final remark, we observe that job satisfaction and on-the-job feedback both have positive impacts on the views of our respondents with regard to the effects of the COVID-19 crisis on future career outcomes. Both variables are related to significantly lower levels of fear regarding job losses, general negative career consequences, declining wages and fewer attractive vacancies. Furthermore, job satisfaction is also related to significantly lower levels of fear with regard to missing out on promotions and less work motivation. Previous research has underlined the positive relationships between the feedback environment (that is, all the relevant cues that provide information about the performance of an individual) and job satisfaction, and between feedback-seeking behaviour and extrinsic (objective) career success measured by wages or promotions [48, 49]. Based on our results, knowing where one stands and how one’s job performance is estimated by others thus appears to have, in addition, a positive influence on self-reported career prospects under the precarious circumstances of the current crisis.

### Perceived evolution in attaching importance to particular job aspects induced by the COVID-19 crisis

**Fig 2:**
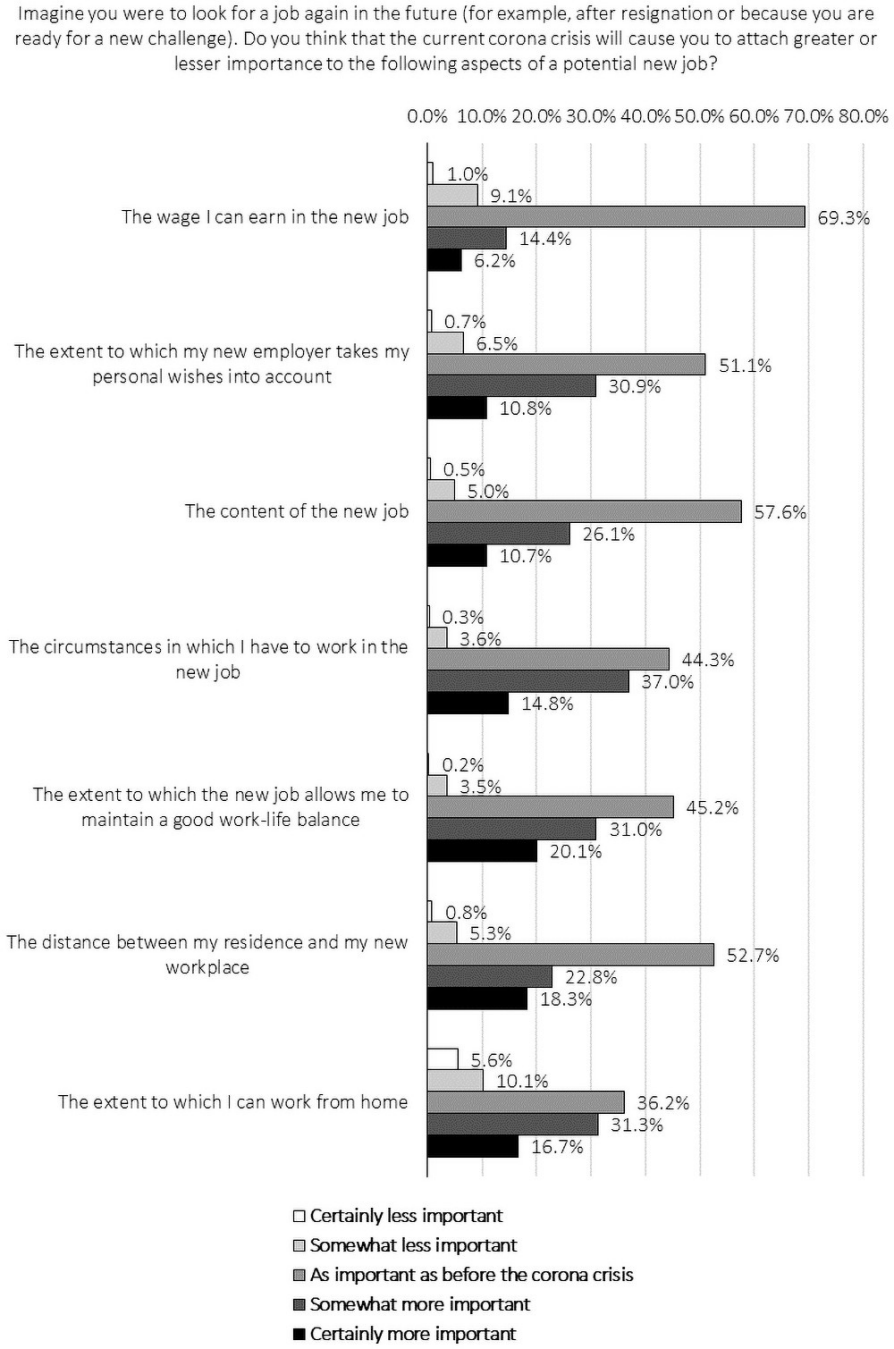
Perceived evolution in attaching importance to particular job aspects induced by the COVID-19 crisis: Answers given.

The survey items relating to RQ2 are analysed by analogy with those discussed in the previous subsection. As can be seen in Fig 2 and Table 3, only about one in five (20.6%) respondents indicate that they will attach more importance to wages due to the COVID-19 crisis, assuming that they were to seek new jobs in the foreseeable future. Conversely, almost seven out of ten (69.3%) participants responded neutrally to the corresponding item. By contrast, more than half indicate to attach more importance to working conditions (51.8%) and work-life balance (51.1%) now, and almost one in two (48.1%) perceive the possibility of teleworking becoming more important following the COVID-19 crisis. Furthermore, just over two out of five attach more importance to the extent to which employers take their personal wishes into account (41.7%), and to the distance from the workplace to home (41.2%).

**Table 3:** Perceived evolution in attaching importance to particular job aspects induced by the COVID-19 crisis: Regression results.

Aspect	% attaching more importance to aspect	Significantly more pronounced if …	Significantly less pronounced if …
Wage	20.6%	More interaction outside of the organisation in job.	Older age; tertiary education; in a relationship and cohabiting; temporarily extended telework; sector is research and development.
Employment relationship	41.7%	More secure contract (public sector or permanent contract in private sector); more interaction outside of the organisation in job; higher % of work potentially done via telework.	Older age; province of East Flanders; living in the centre of a village; better current health; more satisfied with job; more feedback from others in job.
Job content	36.8%	More interaction outside of the organisation in job; sector is education.	In a relationship and cohabiting; more satisfied with job; more feedback from others in job.
Working conditions	51.8%	Sector is health; higher % of work potentially done via telework.	Tertiary education; living in the centre of a city; better current health; more satisfied with job; more feedback from others in job.
Work-life balance	51.1%	Higher number of resident children; COVID-19 patient at the moment (probably); more dependent on others in job; sector is creative, health or education; higher % of work potentially done via telework.	Tertiary education; living in the centre of a village; living in the centre of a city; better current health; more satisfied with job; more autonomous in job; more feedback from others in job.
Distance to the workplace	41.2%	Older age; temporarily unemployed.	Tertiary education; resident others (not family); living in the centre of a village; more satisfied with job; sector is catering and tourism.
Possibility of teleworking	48.1%	Female; better health before the COVID-19 crisis; more secure contract (public sector or permanent contract in private sector); sector is administration, financial or management; temporarily unemployed; higher % of work potentially done via telework; temporarily extended telework.	Older age; living in the centre of a city; better current health; more satisfied with job; more feedback from others in job.

Notes. The proportion ‘attaching more importance’ corresponds to the sum of those who indicated ‘certainly more important’ and ‘somewhat more important’ to the related survey item (see S2 Text in S1 File). The relationship to the personal and job characteristics was analysed by means of a linear regression analysis with heteroscedasticity-robust standard errors (in which all characteristics mentioned in Table 1 were included). The significance level was set as *p* < 0.05. N = 3,821.

With regard to the heterogeneity of the evolution in attached importance to specific job aspects due to the current crisis, we first find that employees who enjoyed tertiary education are less likely to report increased value orientations towards wages, working conditions, work-life balance and distance to the workplace. Considering the heterogeneity in the effects of age on the (changing) importance of job aspects workers value, our results indicate that older workers are less likely to orient themselves towards wage, employment relationships and opportunities to telework, while being more likely to attach increased value to the (commuting) distance to the workplace. Finally, moderation analyses suggest that, due to the crisis, women are more likely than men to pay increased attention to telework possibilities when evaluating a new job opportunity, plausibly due to difficulty of combining greater care-taking responsibilities with job-related possibilities, which can be facilitated by teleworking arrangements [50].

The results are generally in line with previous research that showed that pay is a necessary but not an imperative condition to remain motivated at work, as providing feedback and social recognition also play key roles in this relationship [51]. Social recognition is mainly highlighted by our respondents in terms of contextual factors, such as work-life balance, working conditions and employer rapport (see above), which are allocated more importance to. The sudden changes as a result of the current crisis, including the abrupt introduction of compulsory teleworking for almost all non-essential occupations in Belgium most likely played a substantial role in the increased reports of the importance thereof [52].

## Conclusion

We investigated the expected impact of the COVID-19 crisis on career outcomes and career aspirations via standardised survey research on Flemish employees. We found the fear of negative career impacts due to the COVID-19 crisis to be significant. More than one in four of the respondents in our post-stratified sample indicate to fear losing their jobs because of the crisis. One in seven fear that this will be the case even before the end of the year. It is also striking that one in four fear missing out on a promotion that she/he would have received had the COVID-19 crisis not occurred. The fear of negative impacts is greater in vulnerable groups, such as migrants. In addition, we observed that many respondents believe they will look at the labour market differently and will have different work-related priorities in the future. More than half of the respondents indicate attaching more importance to working conditions and work-life balance since the onset of the COVID-19 crisis.

We end this article with a brief discussion of the limitations of the research. First, as described, we made a great effort to be able to present research results obtained from a relevant group of employees in the short term. Using post-stratification, we ensured that our research sample was representative of our broader research population by using (two levels of) gender, age and educational level. As indicated, we have good reasons to believe that our way of working limited the non-response bias in our results, but we cannot claim that it has been reduced to zero.

Second, the findings cannot be read as an objective prediction of the impact of the COVID-19 crisis on the careers and career aspirations of employees in Flanders, let alone in the Western world as a whole. The results provide an insight into how Flemish employees *think* about this impact *in the midst of this crisis*. In this regard, the extent to which the measured intentions will be realised—or will remain only good intentions, as is often the case with intentions made around New Year’s Eve or after a serious illness—is uncertain. Nevertheless, we believe the mapped fears and perceived evolutions in career aspirations—and heterogeneity both by personal and job characteristics—are relevant to policy makers, as well as to the scientific community. We hope that, based on other research strategies, the latter will reveal whether the outlined fears were justified and whether the expected impact on career aspirations was realised in reality in the medium term.

## Supporting information

S1 File (DOCX)
