# Supplementary material for: How do employees think the COVID-19 crisis will affect their careers?
Source: PLoS One. 2021 May 6;16(5):e0246899. doi: 10.1371/journal.pone.0246899 (PMC8101726; doi:10.1371/journal.pone.0246899)
Supplement: S1 File — (DOCX) [file pone.0246899.s001.docx]

Article title: How do employees think the COVID-19 crisis will affect their careers?

Authors: Lippens, L., Moens, E., Sterkens, P., Weytjens, J., Baert, S.

## S1 Text A. Perceived career-related fears induced by the COVID-19 crisis

The following statements are about how you think the current corona crisis will affect your future career once you have returned to normal work. Please indicate to what extent you agree with the statements on a scale from ‘completely disagree’ (1) to ‘completely agree’ (5).

(Losing job in the short or long term) I am afraid the current corona crisis will cause me to lose my current job in the short or long term.

(Losing job in the short term) I am afraid the current corona crisis will cause me to lose my current job before the end of 2020.

(Missing out on promotion) I am afraid the current corona crisis will result in me not getting a promotion that I would otherwise have received.

(Overall negative impact on career) I am afraid the current corona crisis will have a negative impact on my overall career.

(Negative impact on wage) I am afraid the current corona crisis will have a negative impact on my wage.

(Negative impact on personal motivation) I am afraid the current corona crisis will have a negative impact on my personal work motivation.

(Negative impact on the number of attractive vacancies) I am afraid the current corona crisis will have a negative impact on the number of job vacancies that might interest me.

## S1 Text B. Perceived evolution in attaching importance to particular job aspects induced by the COVID-19 crisis

Imagine you were to look for a job again in the future (for example, after resignation or because you are ready for a new challenge). Do you think that the current corona crisis will cause you to attach greater or lesser importance to the following aspects of a potential new job? Scale: certainly less important (1), somewhat less important (2), as important as before the corona crisis (3), somewhat more important (4), certainly more important (5).

(Wage) The wage I can earn in the new job.

(Employment relationship) The extent to which my new employer takes my personal wishes into account.

(Job content) The content of the new job.

(Working conditions) The circumstances in which I have to work in the new job.

(Work-life balance) The extent to which the new job allows me to maintain a good work-life balance.

(Distance to the workplace) The distance between my residence and my new workplace.

(Possibility of teleworking) The extent to which I can work from home.

| **S1 Table A. Regression Analysis: Fear of Losing Jobs in the Short or Long Term** | | |
| --- | --- | --- |
|  | Linear regression analysis | Ordered logistic regression analysis |
| Female | 0.136*** (0.041) | 0.198*** (0.072) |
| Age | 0.013*** (0.002) | 0.022*** (0.004) |
| Migration background | 0.237** (0.120) | 0.416** (0.192) |
| Tertiary education | 0.020 (0.042) | 0.021 (0.073) |
| Single (reference) |  |  |
| In a relationship but not cohabiting | -0.132* (0.076) | -0.198 (0.133) |
| In a relationship and cohabiting | -0.072 (0.050) | -0.104 (0.085) |
| Number of resident children | -0.023 (0.018) | -0.049 (0.032) |
| Resident parents | 0.033 (0.086) | 0.051 (0.143) |
| Resident family members (other than parents) | -0.072 (0.103) | -0.145 (0.180) |
| Resident others (not family) | 0.090 (0.142) | 0.103 (0.222) |
| Province of Antwerp (reference) |  |  |
| Province of West Flanders | -0.009 (0.053) | 0.023 (0.092) |
| Province of East Flanders | -0.006 (0.046) | -0.004 (0.080) |
| Province of Limburg | 0.055 (0.072) | 0.114 (0.126) |
| Province of Flemish Brabant | 0.112** (0.056) | 0.229** (0.097) |
| Living in the countryside or rural area (reference) |  |  |
| Living in the centre of a village | -0.024 (0.046) | -0.058 (0.079) |
| Living in the suburbs of a city | -0.029 (0.047) | -0.069 (0.083) |
| Living in the centre of a city | -0.029 (0.056) | -0.054 (0.099) |
| Health before the COVID-19 crisis (scale) | 0.027 (0.038) | 0.043 (0.060) |
| Current health (scale) | -0.172*** (0.035) | -0.287*** (0.057) |
| Never been a COVID-19 patient (definitely or likely) (reference) |  |  |
| Uncertain about having been a COVID-19 patient | 0.050 (0.044) | 0.078 (0.076) |
| COVID-19 patient at the moment (definitely or likely) | 0.024 (0.101) | 0.082 (0.169) |
| COVID-19 patient in the recent past (definitely or likely) | 0.093 (0.114) | 0.115 (0.174) |
| Employed via a temporary contract in the private sector (reference) |  |  |
| Employed via a permanent contract in the private sector | -0.650*** (0.109) | -1.021*** (0.165) |
| Employed via a regular contract in the public sector | -1.341*** (0.125) | -2.582*** (0.223) |
| Employed via a permanent appointment in the public sector | -1.042*** (0.122) | -1.680*** (0.204) |
| Part−time contract | 0.004 (0.050) | 0.062 (0.088) |
| Tenure with current employer (scale) | -0.101*** (0.020) | -0.156*** (0.036) |
| Tenure in current job (scale) | -0.001 (0.021) | -0.023 (0.038) |
| Satisfied with job (scale) | -0.109*** (0.022) | -0.213*** (0.038) |
| Autonomous in job (scale) | -0.029* (0.017) | -0.040 (0.029) |
| Dependent on others in job (scale) | 0.037** (0.017) | 0.065** (0.029) |
| Interaction outside of the organisation in job (scale) | 0.018 (0.014) | 0.030 (0.025) |
| Feedback from others in job (scale) | -0.072*** (0.017) | -0.114*** (0.029) |
| Temporarily unemployed | 0.485*** (0.059) | 0.754*** (0.094) |
| % of work potentially done via telework | 0.001 (0.001) | 0.002 (0.001) |
| Temporarily extended telework | 0.024 (0.059) | 0.051 (0.099) |
| Sector: Other (reference) |  |  |
| Sector: Purchasing | 0.171 (0.217) | 0.174 (0.328) |
| Sector: Administration | -0.085 (0.113) | -0.149 (0.180) |
| Sector: Construction | -0.179 (0.131) | -0.272 (0.214) |
| Sector: Communication | -0.113 (0.180) | -0.244 (0.285) |
| Sector: Creative | 0.301 (0.237) | 0.431 (0.354) |
| Sector: Provision of services | -0.283*** (0.106) | -0.456*** (0.176) |
| Sector: Financial | -0.336*** (0.121) | -0.627*** (0.199) |
| Sector: Health | -0.735*** (0.110) | -1.452*** (0.202) |
| Sector: Catering and tourism | 0.625*** (0.142) | 1.014*** (0.219) |
| Sector: Human Resources | -0.046 (0.123) | -0.063 (0.202) |
| Sector: ICT | -0.298*** (0.110) | -0.498*** (0.184) |
| Sector: Legal | -0.283* (0.169) | -0.492* (0.297) |
| Sector: Agriculture and horticulture | -0.893*** (0.254) | -1.435** (0.608) |
| Sector: Logistics and transport | -0.037 (0.112) | -0.113 (0.177) |
| Sector: Management | -0.170 (0.127) | -0.285 (0.207) |
| Sector: Marketing | 0.053 (0.165) | 0.058 (0.267) |
| Sector: Maintenance | -0.319* (0.165) | -0.598** (0.276) |
| Sector: Education | -0.180 (0.127) | -0.280 (0.239) |
| Sector: Research and development | -0.163 (0.148) | -0.385 (0.244) |
| Sector: Government | -0.389*** (0.115) | -0.773*** (0.234) |
| Sector: Production | 0.024 (0.113) | 0.055 (0.183) |
| Sector: Technology | -0.139 (0.126) | -0.231 (0.204) |
| Sector: Sales | 0.149 (0.108) | 0.179 (0.168) |
| N | 3,821 | 3,821 |
| Notes. The presented statistics are coefficient estimates and standard errors in parentheses based on a regression analysis with heteroscedasticity-robust standard errors. Intercepts and cut-off values are not presented. * (**) ((***)) indicates significance at the 10% (5%) ((1%)) level. The significance levels cannot be given an absolute interpretation due to potential multiple testing problems (false positives). | | |

| **S1 Table B. Regression Analysis: Regression analysis: Fear of Losing job in the Short Term** | | |
| --- | --- | --- |
|  | Linear regression analysis | Ordered logistic regression analysis |
| Female | 0.153*** (0.039) | 0.284*** (0.074) |
| Age | 0.018*** (0.002) | 0.031*** (0.004) |
| Migration background | 0.224* (0.115) | 0.426** (0.195) |
| Tertiary education | 0.009 (0.039) | 0.020 (0.076) |
| Single (reference) |  |  |
| In a relationship but not cohabiting | -0.065 (0.073) | -0.109 (0.137) |
| In a relationship and cohabiting | -0.053 (0.046) | -0.084 (0.088) |
| Number of resident children | -0.019 (0.017) | -0.038 (0.033) |
| Resident parents | 0.064 (0.082) | 0.101 (0.148) |
| Resident family members (other than parents) | -0.042 (0.100) | -0.088 (0.187) |
| Resident others (not family) | 0.081 (0.132) | 0.144 (0.224) |
| Province of Antwerp (reference) |  |  |
| Province of West Flanders | -0.036 (0.049) | -0.005 (0.096) |
| Province of East Flanders | -0.016 (0.042) | -0.001 (0.083) |
| Province of Limburg | 0.005 (0.067) | 0.007 (0.131) |
| Province of Flemish Brabant | 0.111** (0.053) | 0.279*** (0.100) |
| Living in the countryside or rural area (reference) |  |  |
| Living in the centre of a village | -0.035 (0.042) | -0.052 (0.081) |
| Living in the suburbs of a city | -0.018 (0.044) | -0.061 (0.087) |
| Living in the centre of a city | -0.029 (0.053) | -0.054 (0.103) |
| Health before the COVID-19 crisis (scale) | 0.025 (0.036) | 0.039 (0.062) |
| Current health (scale) | -0.138*** (0.033) | -0.271*** (0.058) |
| Never been a COVID-19 patient (definitely or likely) (reference) |  |  |
| Uncertain about having been a COVID-19 patient | 0.049 (0.041) | 0.105 (0.078) |
| COVID-19 patient at the moment (definitely or likely) | 0.006 (0.098) | -0.027 (0.179) |
| COVID-19 patient in the recent past (definitely or likely) | 0.105 (0.105) | 0.177 (0.179) |
| Employed via a temporary contract in the private sector (reference) |  |  |
| Employed via a permanent contract in the private sector | -0.662*** (0.110) | -1.004*** (0.163) |
| Employed via a regular contract in the public sector | -1.142*** (0.121) | -2.370*** (0.231) |
| Employed via a permanent appointment in the public sector | -0.965*** (0.121) | -1.650*** (0.209) |
| Part−time contract | 0.004 (0.046) | 0.052 (0.090) |
| Tenure with current employer (scale) | -0.149*** (0.019) | -0.264*** (0.038) |
| Tenure in current job (scale) | 0.005 (0.019) | 0.002 (0.040) |
| Satisfied with job (scale) | -0.097*** (0.021) | -0.211*** (0.039) |
| Autonomous in job (scale) | -0.019 (0.017) | -0.031 (0.030) |
| Dependent on others in job (scale) | 0.029* (0.016) | 0.056* (0.030) |
| Interaction outside of the organisation in job (scale) | 0.001 (0.013) | 0.008 (0.026) |
| Feedback from others in job (scale) | -0.070*** (0.016) | -0.135*** (0.030) |
| Temporarily unemployed | 0.507*** (0.057) | 0.846*** (0.096) |
| % of work potentially done via telework | 0.001** (0.001) | 0.002** (0.001) |
| Temporarily extended telework | -0.047 (0.053) | -0.064 (0.101) |
| Sector: Other (reference) |  |  |
| Sector: Purchasing | 0.040 (0.201) | -0.012 (0.327) |
| Sector: Administration | -0.086 (0.108) | -0.139 (0.184) |
| Sector: Construction | -0.108 (0.126) | -0.181 (0.222) |
| Sector: Communication | -0.017 (0.173) | -0.102 (0.296) |
| Sector: Creative | -0.098 (0.227) | -0.159 (0.372) |
| Sector: Provision of services | -0.227** (0.101) | -0.382** (0.181) |
| Sector: Financial | -0.240** (0.113) | -0.484** (0.205) |
| Sector: Health | -0.665*** (0.101) | -1.545*** (0.217) |
| Sector: Catering and tourism | 0.502*** (0.142) | 0.844*** (0.219) |
| Sector: Human Resources | 0.002 (0.116) | 0.062 (0.205) |
| Sector: ICT | -0.209** (0.105) | -0.364* (0.190) |
| Sector: Legal | -0.148 (0.158) | -0.234 (0.305) |
| Sector: Agriculture and horticulture | -0.584*** (0.217) | -0.969 (0.612) |
| Sector: Logistics and transport | -0.029 (0.107) | -0.055 (0.181) |
| Sector: Management | -0.128 (0.118) | -0.204 (0.212) |
| Sector: Marketing | 0.073 (0.154) | 0.151 (0.270) |
| Sector: Maintenance | -0.316** (0.140) | -0.501* (0.280) |
| Sector: Education | -0.299*** (0.114) | -0.736*** (0.266) |
| Sector: Research and development | -0.132 (0.134) | -0.236 (0.250) |
| Sector: Government | -0.327*** (0.106) | -0.795*** (0.255) |
| Sector: Production | -0.043 (0.105) | 0.031 (0.187) |
| Sector: Technology | -0.139 (0.115) | -0.159 (0.208) |
| Sector: Sales | 0.241** (0.105) | 0.389** (0.171) |
| N | 3,821 | 3,821 |
| Notes. The presented statistics are coefficient estimates and standard errors in parentheses based on a regression analysis with heteroscedasticity-robust standard errors. Intercepts and cut-off values are not presented. * (**) ((***)) indicates significance at the 10% (5%) ((1%)) level. The significance levels cannot be given an absolute interpretation due to potential multiple testing problems (false positives). | | |

| **S1 Table C. Regression Analysis: Fear of Negative Impact on Wage** | | |
| --- | --- | --- |
|  | Linear regression analysis | Ordered logistic regression analysis |
| Female | -0.053 (0.048) | -0.071 (0.069) |
| Age | -0.004 (0.002) | -0.006* (0.004) |
| Migration background | -0.101 (0.130) | -0.147 (0.184) |
| Tertiary education | -0.020 (0.049) | -0.032 (0.071) |
| Single (reference) |  |  |
| In a relationship but not cohabiting | -0.021 (0.088) | -0.030 (0.129) |
| In a relationship and cohabiting | -0.144** (0.057) | -0.235*** (0.082) |
| Number of resident children | 0.071*** (0.020) | 0.111*** (0.030) |
| Resident parents | -0.099 (0.097) | -0.156 (0.138) |
| Resident family members (other than parents) | -0.083 (0.118) | -0.086 (0.176) |
| Resident others (not family) | -0.257* (0.156) | -0.360* (0.217) |
| Province of Antwerp (reference) |  |  |
| Province of West Flanders | 0.153** (0.061) | 0.212** (0.089) |
| Province of East Flanders | 0.097* (0.053) | 0.141* (0.077) |
| Province of Limburg | 0.125 (0.083) | 0.188 (0.121) |
| Province of Flemish Brabant | 0.073 (0.066) | 0.098 (0.095) |
| Living in the countryside or rural area (reference) |  |  |
| Living in the centre of a village | -0.049 (0.053) | -0.073 (0.076) |
| Living in the suburbs of a city | 0.042 (0.055) | 0.077 (0.080) |
| Living in the centre of a city | -0.027 (0.066) | -0.029 (0.096) |
| Health before the COVID-19 crisis (scale) | 0.067 (0.041) | 0.109* (0.060) |
| Current health (scale) | -0.205*** (0.039) | -0.305*** (0.057) |
| Never been a COVID-19 patient (definitely or likely) (reference) |  |  |
| Uncertain about having been a COVID-19 patient | 0.016 (0.050) | 0.026 (0.073) |
| COVID-19 patient at the moment (definitely or likely) | -0.018 (0.116) | 0.049 (0.169) |
| COVID-19 patient in the recent past (definitely or likely) | 0.279** (0.114) | 0.411** (0.168) |
| Employed via a temporary contract in the private sector (reference) |  |  |
| Employed via a permanent contract in the private sector | 0.064 (0.108) | 0.091 (0.158) |
| Employed via a regular contract in the public sector | -0.609*** (0.142) | -0.895*** (0.204) |
| Employed via a permanent appointment in the public sector | -0.663*** (0.132) | -0.911*** (0.193) |
| Part−time contract | 0.036 (0.059) | 0.055 (0.086) |
| Tenure with current employer (scale) | 0.028 (0.024) | 0.043 (0.035) |
| Tenure in current job (scale) | -0.044* (0.026) | -0.063* (0.037) |
| Satisfied with job (scale) | -0.063** (0.026) | -0.082** (0.037) |
| Autonomous in job (scale) | -0.015 (0.020) | -0.021 (0.029) |
| Dependent on others in job (scale) | 0.028 (0.019) | 0.039 (0.028) |
| Interaction outside of the organisation in job (scale) | 0.039** (0.017) | 0.061** (0.024) |
| Feedback from others in job (scale) | -0.073*** (0.020) | -0.112*** (0.028) |
| Temporarily unemployed | 0.415*** (0.063) | 0.605*** (0.093) |
| % of work potentially done via telework | -0.000 (0.001) | -0.001 (0.001) |
| Temporarily extended telework | 0.072 (0.067) | 0.118 (0.095) |
| Sector: Other (reference) |  |  |
| Sector: Purchasing | 0.307 (0.195) | 0.407 (0.313) |
| Sector: Administration | -0.168 (0.127) | -0.246 (0.178) |
| Sector: Construction | -0.070 (0.145) | -0.114 (0.211) |
| Sector: Communication | -0.094 (0.205) | -0.123 (0.280) |
| Sector: Creative | -0.174 (0.280) | -0.276 (0.376) |
| Sector: Provision of services | -0.032 (0.121) | -0.053 (0.173) |
| Sector: Financial | -0.172 (0.138) | -0.264 (0.193) |
| Sector: Health | -0.639*** (0.132) | -0.890*** (0.188) |
| Sector: Catering and tourism | 0.356** (0.140) | 0.499** (0.216) |
| Sector: Human Resources | 0.093 (0.140) | 0.116 (0.200) |
| Sector: ICT | -0.075 (0.130) | -0.094 (0.183) |
| Sector: Legal | 0.089 (0.193) | 0.087 (0.284) |
| Sector: Agriculture and horticulture | -0.235 (0.338) | -0.374 (0.534) |
| Sector: Logistics and transport | -0.006 (0.123) | -0.035 (0.175) |
| Sector: Management | 0.121 (0.143) | 0.183 (0.204) |
| Sector: Marketing | 0.190 (0.174) | 0.192 (0.261) |
| Sector: Maintenance | 0.046 (0.177) | 0.028 (0.262) |
| Sector: Education | -0.229 (0.156) | -0.344 (0.222) |
| Sector: Research and development | -0.175 (0.168) | -0.217 (0.234) |
| Sector: Government | -0.347** (0.154) | -0.539** (0.218) |
| Sector: Production | -0.096 (0.130) | -0.172 (0.183) |
| Sector: Technology | 0.041 (0.143) | 0.043 (0.203) |
| Sector: Sales | 0.088 (0.116) | 0.093 (0.167) |
| N | 3,821 | 3,821 |
| Notes. The presented statistics are coefficient estimates and standard errors in parentheses based on a regression analysis with heteroscedasticity-robust standard errors. Intercepts and cut-off values are not presented. * (**) ((***)) indicates significance at the 10% (5%) ((1%)) level. The significance levels cannot be given an absolute interpretation due to potential multiple testing problems (false positives). | | |

| **S1 Table D. Regression Analysis: Fear of Missing Out on Promotion** | | |
| --- | --- | --- |
|  | Linear regression analysis | Ordered logistic regression analysis |
| Female | 0.071 (0.047) | 0.093 (0.070) |
| Age | -0.006** (0.002) | -0.009*** (0.004) |
| Migration background | 0.272** (0.122) | 0.392** (0.182) |
| Tertiary education | -0.111** (0.048) | -0.173** (0.071) |
| Single (reference) |  |  |
| In a relationship but not cohabiting | 0.019 (0.085) | 0.028 (0.127) |
| In a relationship and cohabiting | 0.035 (0.055) | 0.064 (0.082) |
| Number of resident children | 0.014 (0.021) | 0.020 (0.030) |
| Resident parents | -0.054 (0.094) | -0.093 (0.137) |
| Resident family members (other than parents) | 0.052 (0.120) | 0.024 (0.175) |
| Resident others (not family) | 0.059 (0.136) | 0.070 (0.208) |
| Province of Antwerp (reference) |  |  |
| Province of West Flanders | 0.084 (0.060) | 0.120 (0.089) |
| Province of East Flanders | 0.050 (0.052) | 0.075 (0.077) |
| Province of Limburg | 0.000 (0.079) | 0.001 (0.120) |
| Province of Flemish Brabant | 0.165** (0.065) | 0.226** (0.095) |
| Living in the countryside or rural area (reference) |  |  |
| Living in the centre of a village | 0.029 (0.052) | 0.045 (0.077) |
| Living in the suburbs of a city | 0.044 (0.054) | 0.083 (0.080) |
| Living in the centre of a city | -0.000 (0.064) | -0.014 (0.095) |
| Health before the COVID-19 crisis (scale) | 0.076* (0.040) | 0.112* (0.058) |
| Current health (scale) | -0.134*** (0.038) | -0.204*** (0.055) |
| Never been a COVID-19 patient (definitely or likely) (reference) |  |  |
| Uncertain about having been a COVID-19 patient | 0.003 (0.049) | 0.007 (0.073) |
| COVID-19 patient at the moment (definitely or likely) | 0.100 (0.113) | 0.150 (0.166) |
| COVID-19 patient in the recent past (definitely or likely) | 0.123 (0.120) | 0.186 (0.169) |
| Employed via a temporary contract in the private sector (reference) |  |  |
| Employed via a permanent contract in the private sector | -0.100 (0.107) | -0.161 (0.155) |
| Employed via a regular contract in the public sector | -0.622*** (0.133) | -1.019*** (0.202) |
| Employed via a permanent appointment in the public sector | -0.468*** (0.128) | -0.644*** (0.191) |
| Part−time contract | -0.186*** (0.056) | -0.255*** (0.086) |
| Tenure with current employer (scale) | -0.019 (0.024) | -0.028 (0.035) |
| Tenure in current job (scale) | -0.022 (0.025) | -0.025 (0.037) |
| Satisfied with job (scale) | -0.101*** (0.025) | -0.160*** (0.037) |
| Autonomous in job (scale) | -0.021 (0.020) | -0.027 (0.028) |
| Dependent on others in job (scale) | 0.028 (0.019) | 0.041 (0.028) |
| Interaction outside of the organisation in job (scale) | 0.048*** (0.016) | 0.072*** (0.024) |
| Feedback from others in job (scale) | -0.022 (0.020) | -0.022 (0.028) |
| Sector: Purchasing | 0.250*** (0.064) | 0.350*** (0.092) |
| Sector: Administration | 0.001 (0.001) | 0.002* (0.001) |
| Sector: Construction | 0.043 (0.064) | 0.053 (0.095) |
| Sector: Communication |  |  |
| Sector: Creative | 0.160 (0.217) | 0.194 (0.315) |
| Sector: Provision of services | 0.183 (0.118) | 0.249 (0.175) |
| Sector: Financial | 0.228 (0.145) | 0.301 (0.210) |
| Sector: Health | 0.241 (0.193) | 0.340 (0.275) |
| Sector: Catering and tourism | 0.165 (0.255) | 0.179 (0.354) |
| Sector: Human Resources | 0.094 (0.114) | 0.114 (0.171) |
| Sector: ICT | 0.249* (0.128) | 0.330* (0.189) |
| Sector: Legal | -0.319*** (0.120) | -0.571*** (0.191) |
| Sector: Agriculture and horticulture | 0.355** (0.140) | 0.472** (0.207) |
| Sector: Logistics and transport | 0.268** (0.133) | 0.370* (0.197) |
| Sector: Management | 0.254** (0.120) | 0.343* (0.178) |
| Sector: Marketing | 0.522*** (0.173) | 0.758*** (0.274) |
| Sector: Maintenance | 0.218 (0.352) | 0.358 (0.539) |
| Sector: Education | 0.241** (0.116) | 0.319* (0.172) |
| Sector: Research and development | 0.110 (0.135) | 0.134 (0.200) |
| Sector: Government | 0.357* (0.190) | 0.493* (0.267) |
| Sector: Production | 0.126 (0.179) | 0.213 (0.259) |
| Sector: Technology | -0.071 (0.137) | -0.175 (0.224) |
| Sector: Sales | 0.186 (0.152) | 0.242 (0.231) |
| Sector: Other (reference) | -0.144 (0.135) | -0.275 (0.217) |
| Temporarily unemployed | 0.271** (0.115) | 0.386** (0.176) |
| % of work potentially done via telework | 0.349*** (0.134) | 0.488** (0.198) |
| Temporarily extended telework | 0.145 (0.109) | 0.189 (0.162) |
| N | 3,821 | 3,821 |
| Notes. The presented statistics are coefficient estimates and standard errors in parentheses based on a regression analysis with heteroscedasticity-robust standard errors. Intercepts and cut-off values are not presented. * (**) ((***)) indicates significance at the 10% (5%) ((1%)) level. The significance levels cannot be given an absolute interpretation due to potential multiple testing problems (false positives). | | |

| **S1 Table E. Regression Analysis:Fear oF Negative Impact on the Number of Attractive** | | |
| --- | --- | --- |
|  | Linear regression analysis | Ordered logistic regression analysis |
| Female | -0.087** (0.044) | -0.137** (0.069) |
| Age | -0.005** (0.002) | -0.008** (0.004) |
| Migration background | -0.066 (0.127) | -0.099 (0.189) |
| Tertiary education | 0.165*** (0.046) | 0.270*** (0.071) |
| Single (reference) |  |  |
| In a relationship but not cohabiting | -0.066 (0.081) | -0.113 (0.128) |
| In a relationship and cohabiting | -0.005 (0.052) | 0.005 (0.081) |
| Number of resident children | -0.008 (0.019) | -0.022 (0.030) |
| Resident parents | 0.094 (0.085) | 0.122 (0.137) |
| Resident family members (other than parents) | -0.171 (0.107) | -0.273 (0.174) |
| Resident others (not family) | 0.001 (0.128) | 0.015 (0.211) |
| Province of Antwerp (reference) |  |  |
| Province of West Flanders | 0.115** (0.056) | 0.186** (0.089) |
| Province of East Flanders | 0.112** (0.049) | 0.168** (0.077) |
| Province of Limburg | 0.063 (0.082) | 0.138 (0.123) |
| Province of Flemish Brabant | 0.117* (0.060) | 0.168* (0.095) |
| Living in the countryside or rural area (reference) |  |  |
| Living in the centre of a village | -0.050 (0.049) | -0.079 (0.076) |
| Living in the suburbs of a city | 0.018 (0.051) | 0.020 (0.080) |
| Living in the centre of a city | 0.062 (0.060) | 0.096 (0.096) |
| Health before the COVID-19 crisis (scale) | 0.114*** (0.039) | 0.205*** (0.060) |
| Current health (scale) | -0.149*** (0.036) | -0.239*** (0.056) |
| Never been a COVID-19 patient (definitely or likely) (reference) |  |  |
| Uncertain about having been a COVID-19 patient | 0.032 (0.047) | 0.054 (0.073) |
| COVID-19 patient at the moment (definitely or likely) | -0.106 (0.109) | -0.135 (0.168) |
| COVID-19 patient in the recent past (definitely or likely) | 0.060 (0.111) | 0.086 (0.170) |
| Employed via a temporary contract in the private sector (reference) |  |  |
| Employed via a permanent contract in the private sector | -0.168* (0.100) | -0.356** (0.160) |
| Employed via a regular contract in the public sector | -0.541*** (0.131) | -0.931*** (0.204) |
| Employed via a permanent appointment in the public sector | -0.295** (0.124) | -0.569*** (0.195) |
| Part−time contract | 0.043 (0.054) | 0.057 (0.085) |
| Tenure with current employer (scale) | -0.021 (0.022) | -0.048 (0.035) |
| Tenure in current job (scale) | -0.038 (0.024) | -0.054 (0.037) |
| Satisfied with job (scale) | -0.209*** (0.023) | -0.358*** (0.038) |
| Autonomous in job (scale) | -0.004 (0.019) | -0.002 (0.029) |
| Dependent on others in job (scale) | 0.040** (0.018) | 0.079*** (0.028) |
| Interaction outside of the organisation in job (scale) | 0.015 (0.016) | 0.022 (0.024) |
| Feedback from others in job (scale) | -0.042** (0.018) | -0.072** (0.028) |
| Sector: Purchasing | 0.160*** (0.058) | 0.253*** (0.093) |
| Sector: Administration | 0.001* (0.001) | 0.002** (0.001) |
| Sector: Construction | 0.005 (0.060) | -0.031 (0.095) |
| Sector: Communication |  |  |
| Sector: Creative | -0.262 (0.202) | -0.424 (0.320) |
| Sector: Provision of services | -0.054 (0.109) | -0.077 (0.175) |
| Sector: Financial | -0.245* (0.134) | -0.394* (0.212) |
| Sector: Health | 0.161 (0.148) | 0.217 (0.266) |
| Sector: Catering and tourism | 0.572*** (0.181) | 0.946*** (0.359) |
| Sector: Human Resources | -0.193* (0.107) | -0.315* (0.171) |
| Sector: ICT | -0.065 (0.120) | -0.056 (0.191) |
| Sector: Legal | -0.488*** (0.122) | -0.722*** (0.189) |
| Sector: Agriculture and horticulture | 0.174 (0.132) | 0.327 (0.215) |
| Sector: Logistics and transport | 0.076 (0.119) | 0.107 (0.198) |
| Sector: Management | -0.233** (0.113) | -0.336* (0.179) |
| Sector: Marketing | -0.287* (0.160) | -0.459* (0.271) |
| Sector: Maintenance | -0.732** (0.367) | -1.136** (0.573) |
| Sector: Education | 0.118 (0.105) | 0.152 (0.172) |
| Sector: Research and development | -0.004 (0.133) | 0.053 (0.205) |
| Sector: Government | 0.298** (0.143) | 0.458* (0.265) |
| Sector: Production | -0.429** (0.169) | -0.624** (0.260) |
| Sector: Technology | -0.265* (0.147) | -0.329 (0.222) |
| Sector: Sales | -0.005 (0.149) | 0.055 (0.235) |
| Sector: Other (reference) | -0.260* (0.140) | -0.328 (0.213) |
| Temporarily unemployed | -0.037 (0.113) | -0.096 (0.180) |
| % of work potentially done via telework | -0.125 (0.131) | -0.184 (0.201) |
| Temporarily extended telework | -0.017 (0.100) | -0.044 (0.163) |
| N | 3,821 | 3,821 |
| Notes. The presented statistics are coefficient estimates and standard errors in parentheses based on a regression analysis with heteroscedasticity-robust standard errors. Intercepts and cut-off values are not presented. * (**) ((***)) indicates significance at the 10% (5%) ((1%)) level. The significance levels cannot be given an absolute interpretation due to potential multiple testing problems (false positives). | | |

| **S1 Table F. Regression Analysis: Fear of Negative Impact on Personal Motivation** | | |
| --- | --- | --- |
|  | Linear regression analysis | Ordered logistic regression analysis |
| Female | 0.044 (0.045) | 0.087 (0.070) |
| Age | -0.013*** (0.002) | -0.021*** (0.004) |
| Migration background | 0.007 (0.120) | -0.000 (0.187) |
| Tertiary education | -0.019 (0.046) | -0.028 (0.072) |
| Single (reference) |  |  |
| In a relationship but not cohabiting | 0.097 (0.082) | 0.160 (0.128) |
| In a relationship and cohabiting | 0.035 (0.055) | 0.050 (0.083) |
| Number of resident children | -0.007 (0.020) | -0.015 (0.031) |
| Resident parents | -0.017 (0.089) | -0.018 (0.138) |
| Resident family members (other than parents) | -0.059 (0.111) | -0.078 (0.177) |
| Resident others (not family) | -0.037 (0.142) | -0.055 (0.219) |
| Province of Antwerp (reference) |  |  |
| Province of West Flanders | 0.017 (0.058) | 0.040 (0.090) |
| Province of East Flanders | -0.004 (0.050) | -0.013 (0.078) |
| Province of Limburg | -0.079 (0.077) | -0.102 (0.123) |
| Province of Flemish Brabant | 0.033 (0.060) | 0.083 (0.095) |
| Living in the countryside or rural area (reference) |  |  |
| Living in the centre of a village | -0.052 (0.049) | -0.093 (0.077) |
| Living in the suburbs of a city | 0.116** (0.051) | 0.187** (0.081) |
| Living in the centre of a city | 0.037 (0.064) | 0.059 (0.097) |
| Health before the COVID-19 crisis (scale) | 0.137*** (0.041) | 0.204*** (0.060) |
| Current health (scale) | -0.337*** (0.039) | -0.530*** (0.057) |
| Never been a COVID-19 patient (definitely or likely) (reference) |  |  |
| Uncertain about having been a COVID-19 patient | 0.024 (0.047) | 0.026 (0.074) |
| COVID-19 patient at the moment (definitely or likely) | -0.221* (0.122) | -0.377** (0.175) |
| COVID-19 patient in the recent past (definitely or likely) | -0.105 (0.112) | -0.108 (0.167) |
| Employed via a temporary contract in the private sector (reference) |  |  |
| Employed via a permanent contract in the private sector | 0.143 (0.098) | 0.241 (0.156) |
| Employed via a regular contract in the public sector | 0.068 (0.127) | 0.104 (0.203) |
| Employed via a permanent appointment in the public sector | 0.069 (0.123) | 0.102 (0.193) |
| Part−time contract | 0.007 (0.055) | 0.041 (0.086) |
| Tenure with current employer (scale) | -0.013 (0.023) | -0.014 (0.036) |
| Tenure in current job (scale) | 0.038 (0.024) | 0.050 (0.038) |
| Satisfied with job (scale) | -0.419*** (0.025) | -0.676*** (0.039) |
| Autonomous in job (scale) | -0.017 (0.019) | -0.024 (0.029) |
| Dependent on others in job (scale) | 0.042** (0.019) | 0.060** (0.028) |
| Interaction outside of the organisation in job (scale) | 0.025 (0.016) | 0.040* (0.024) |
| Feedback from others in job (scale) | -0.035* (0.019) | -0.052* (0.029) |
| Sector: Purchasing | -0.028 (0.061) | -0.072 (0.093) |
| Sector: Administration | 0.001* (0.001) | 0.002* (0.001) |
| Sector: Construction | -0.018 (0.061) | -0.042 (0.095) |
| Sector: Communication |  |  |
| Sector: Creative | -0.341* (0.200) | -0.527 (0.329) |
| Sector: Provision of services | -0.155 (0.118) | -0.208 (0.180) |
| Sector: Financial | 0.009 (0.138) | 0.101 (0.212) |
| Sector: Health | 0.281 (0.178) | 0.524* (0.272) |
| Sector: Catering and tourism | -0.252 (0.241) | -0.337 (0.354) |
| Sector: Human Resources | -0.047 (0.115) | -0.014 (0.175) |
| Sector: ICT | -0.092 (0.122) | -0.043 (0.193) |
| Sector: Legal | -0.001 (0.124) | -0.011 (0.191) |
| Sector: Agriculture and horticulture | -0.047 (0.136) | -0.052 (0.214) |
| Sector: Logistics and transport | -0.126 (0.126) | -0.100 (0.199) |
| Sector: Management | 0.013 (0.121) | 0.074 (0.183) |
| Sector: Marketing | 0.020 (0.177) | 0.187 (0.280) |
| Sector: Maintenance | 0.131 (0.293) | 0.365 (0.525) |
| Sector: Education | -0.099 (0.113) | -0.086 (0.175) |
| Sector: Research and development | -0.081 (0.136) | -0.040 (0.207) |
| Sector: Government | 0.234 (0.184) | 0.393 (0.268) |
| Sector: Production | -0.050 (0.152) | 0.003 (0.257) |
| Sector: Technology | -0.047 (0.152) | 0.017 (0.230) |
| Sector: Sales | 0.309* (0.160) | 0.515** (0.238) |
| Sector: Other (reference) | -0.150 (0.141) | -0.151 (0.218) |
| Temporarily unemployed | 0.015 (0.120) | 0.145 (0.184) |
| % of work potentially done via telework | 0.083 (0.135) | 0.202 (0.204) |
| Temporarily extended telework | -0.030 (0.112) | -0.006 (0.169) |
| N | 3,821 | 3,821 |
| Notes. The presented statistics are coefficient estimates and standard errors in parentheses based on a regression analysis with heteroscedasticity-robust standard errors. Intercepts and cut-off values are not presented. * (**) ((***)) indicates significance at the 10% (5%) ((1%)) level. The significance levels cannot be given an absolute interpretation due to potential multiple testing problems (false positives). | | |

| **S1 Table G. Regression Analysis: Fear of Overall Negative Impact on Career** | | |
| --- | --- | --- |
|  | Linear regression analysis | Ordered logistic regression analysis |
| Female | 0.094** (0.045) | 0.162** (0.069) |
| Age | 0.007*** (0.002) | 0.011*** (0.004) |
| Migration background | -0.118 (0.131) | -0.245 (0.192) |
| Tertiary education | 0.012 (0.045) | 0.018 (0.071) |
| Single (reference) |  |  |
| In a relationship but not cohabiting | -0.141* (0.081) | -0.179 (0.128) |
| In a relationship and cohabiting | -0.040 (0.054) | -0.042 (0.083) |
| Number of resident children | -0.021 (0.019) | -0.030 (0.030) |
| Resident parents | 0.034 (0.091) | 0.051 (0.138) |
| Resident family members (other than parents) | 0.041 (0.109) | 0.032 (0.175) |
| Resident others (not family) | 0.006 (0.137) | -0.032 (0.212) |
| Province of Antwerp (reference) |  |  |
| Province of West Flanders | 0.141** (0.057) | 0.238*** (0.089) |
| Province of East Flanders | 0.055 (0.050) | 0.101 (0.078) |
| Province of Limburg | 0.087 (0.079) | 0.114 (0.123) |
| Province of Flemish Brabant | 0.176*** (0.060) | 0.285*** (0.094) |
| Living in the countryside or rural area (reference) |  |  |
| Living in the centre of a village | -0.031 (0.049) | -0.039 (0.076) |
| Living in the suburbs of a city | -0.009 (0.051) | -0.015 (0.080) |
| Living in the centre of a city | 0.021 (0.063) | 0.015 (0.096) |
| Health before the COVID-19 crisis (scale) | 0.093** (0.039) | 0.148** (0.059) |
| Current health (scale) | -0.227*** (0.037) | -0.358*** (0.056) |
| Never been a COVID-19 patient (definitely or likely) (reference) |  |  |
| Uncertain about having been a COVID-19 patient | 0.014 (0.047) | 0.020 (0.073) |
| COVID-19 patient at the moment (definitely or likely) | -0.056 (0.111) | -0.035 (0.168) |
| COVID-19 patient in the recent past (definitely or likely) | 0.147 (0.108) | 0.205 (0.167) |
| Employed via a temporary contract in the private sector (reference) |  |  |
| Employed via a permanent contract in the private sector | -0.329*** (0.102) | -0.522*** (0.159) |
| Employed via a regular contract in the public sector | -0.749*** (0.126) | -1.197*** (0.203) |
| Employed via a permanent appointment in the public sector | -0.540*** (0.123) | -0.791*** (0.194) |
| Part−time contract | 0.009 (0.054) | 0.015 (0.085) |
| Tenure with current employer (scale) | -0.048** (0.023) | -0.076** (0.035) |
| Tenure in current job (scale) | 0.006 (0.024) | 0.005 (0.037) |
| Satisfied with job (scale) | -0.113*** (0.024) | -0.191*** (0.037) |
| Autonomous in job (scale) | -0.029 (0.019) | -0.045 (0.029) |
| Dependent on others in job (scale) | 0.048*** (0.018) | 0.077*** (0.028) |
| Interaction outside of the organisation in job (scale) | 0.043*** (0.015) | 0.066*** (0.024) |
| Feedback from others in job (scale) | -0.066*** (0.019) | -0.100*** (0.028) |
| Sector: Purchasing | 0.457*** (0.061) | 0.677*** (0.093) |
| Sector: Administration | -0.000 (0.001) | -0.000 (0.001) |
| Sector: Construction | 0.017 (0.062) | 0.024 (0.095) |
| Sector: Communication |  |  |
| Sector: Creative | -0.139 (0.214) | -0.274 (0.322) |
| Sector: Provision of services | -0.035 (0.117) | -0.074 (0.176) |
| Sector: Financial | -0.065 (0.138) | -0.087 (0.210) |
| Sector: Health | 0.310* (0.176) | 0.458* (0.271) |
| Sector: Catering and tourism | 0.172 (0.265) | 0.245 (0.373) |
| Sector: Human Resources | -0.198* (0.113) | -0.327* (0.172) |
| Sector: ICT | -0.140 (0.130) | -0.259 (0.195) |
| Sector: Legal | -0.542*** (0.120) | -0.925*** (0.188) |
| Sector: Agriculture and horticulture | 0.513*** (0.146) | 0.908*** (0.220) |
| Sector: Logistics and transport | 0.130 (0.127) | 0.160 (0.197) |
| Sector: Management | -0.141 (0.117) | -0.205 (0.179) |
| Sector: Marketing | 0.097 (0.182) | 0.140 (0.281) |
| Sector: Maintenance | 0.258 (0.363) | 0.486 (0.576) |
| Sector: Education | -0.045 (0.116) | -0.110 (0.174) |
| Sector: Research and development | -0.066 (0.135) | -0.114 (0.203) |
| Sector: Government | 0.156 (0.169) | 0.223 (0.264) |
| Sector: Production | -0.299* (0.169) | -0.453* (0.259) |
| Sector: Technology | -0.216 (0.139) | -0.372* (0.222) |
| Sector: Sales | -0.086 (0.151) | -0.166 (0.233) |
| Sector: Other (reference) | -0.429*** (0.130) | -0.696*** (0.214) |
| Temporarily unemployed | 0.102 (0.122) | 0.121 (0.182) |
| % of work potentially done via telework | -0.001 (0.132) | -0.024 (0.200) |
| Temporarily extended telework | 0.186* (0.111) | 0.258 (0.166) |
| N | 3,821 | 3,821 |
| Notes. The presented statistics are coefficient estimates and standard errors in parentheses based on a regression analysis with heteroscedasticity-robust standard errors. Intercepts and cut-off values are not presented. * (**) ((***)) indicates significance at the 10% (5%) ((1%)) level. The significance levels cannot be given an absolute interpretation due to potential multiple testing problems (false positives). | | |

| **S1 Table H. Regression Analysis: Evolution in Attaching Importance to Job Content** | | |
| --- | --- | --- |
|  | Linear regression analysis | Ordered logistic regression analysis |
| Female | -0.024 (0.029) | -0.085 (0.076) |
| Age | -0.001 (0.001) | 0.000 (0.004) |
| Migration background | -0.044 (0.082) | -0.104 (0.207) |
| Tertiary education | -0.037 (0.030) | -0.092 (0.077) |
| Single (reference) |  |  |
| In a relationship but not cohabiting | -0.017 (0.055) | -0.033 (0.140) |
| In a relationship and cohabiting | -0.072** (0.034) | -0.155* (0.089) |
| Number of resident children | -0.007 (0.013) | -0.021 (0.033) |
| Resident parents | -0.021 (0.062) | -0.042 (0.152) |
| Resident family members (other than parents) | -0.042 (0.073) | -0.102 (0.192) |
| Resident others (not family) | -0.099 (0.088) | -0.198 (0.230) |
| Province of Antwerp (reference) |  |  |
| Province of West Flanders | 0.048 (0.038) | 0.148 (0.097) |
| Province of East Flanders | 0.004 (0.032) | 0.033 (0.084) |
| Province of Limburg | 0.084* (0.050) | 0.200 (0.130) |
| Province of Flemish Brabant | 0.003 (0.038) | 0.032 (0.103) |
| Living in the countryside or rural area (reference) |  |  |
| Living in the centre of a village | -0.060* (0.032) | -0.156* (0.083) |
| Living in the suburbs of a city | -0.037 (0.034) | -0.088 (0.087) |
| Living in the centre of a city | -0.064 (0.040) | -0.176* (0.104) |
| Health before the COVID-19 crisis (scale) | 0.018 (0.029) | 0.027 (0.065) |
| Current health (scale) | -0.038 (0.026) | -0.091 (0.061) |
| Never been a COVID-19 patient (definitely or likely) (reference) |  |  |
| Uncertain about having been a COVID-19 patient | 0.007 (0.031) | -0.010 (0.080) |
| COVID-19 patient at the moment (definitely or likely) | -0.045 (0.076) | -0.101 (0.183) |
| COVID-19 patient in the recent past (definitely or likely) | -0.054 (0.070) | -0.077 (0.182) |
| Employed via a temporary contract in the private sector (reference) |  |  |
| Employed via a permanent contract in the private sector | 0.098 (0.063) | 0.243 (0.172) |
| Employed via a regular contract in the public sector | 0.135* (0.082) | 0.349 (0.219) |
| Employed via a permanent appointment in the public sector | 0.075 (0.077) | 0.181 (0.210) |
| Part−time contract | 0.037 (0.034) | 0.118 (0.091) |
| Tenure with current employer (scale) | 0.021 (0.015) | 0.062 (0.038) |
| Tenure in current job (scale) | -0.009 (0.016) | -0.029 (0.040) |
| Satisfied with job (scale) | -0.096*** (0.017) | -0.267*** (0.040) |
| Autonomous in job (scale) | 0.002 (0.012) | 0.011 (0.031) |
| Dependent on others in job (scale) | 0.000 (0.012) | 0.004 (0.030) |
| Interaction outside of the organisation in job (scale) | 0.026*** (0.010) | 0.067*** (0.026) |
| Feedback from others in job (scale) | -0.031** (0.012) | -0.078** (0.031) |
| Sector: Purchasing | -0.017 (0.040) | -0.010 (0.100) |
| Sector: Administration | 0.001 (0.001) | 0.002 (0.001) |
| Sector: Construction | -0.049 (0.040) | -0.148 (0.103) |
| Sector: Communication |  |  |
| Sector: Creative | -0.067 (0.131) | -0.304 (0.359) |
| Sector: Provision of services | 0.017 (0.074) | 0.029 (0.193) |
| Sector: Financial | -0.016 (0.089) | 0.021 (0.231) |
| Sector: Health | -0.113 (0.108) | -0.161 (0.300) |
| Sector: Catering and tourism | 0.081 (0.162) | 0.071 (0.399) |
| Sector: Human Resources | 0.053 (0.071) | 0.144 (0.187) |
| Sector: ICT | 0.014 (0.078) | 0.039 (0.210) |
| Sector: Legal | 0.126* (0.075) | 0.305 (0.201) |
| Sector: Agriculture and horticulture | 0.035 (0.090) | 0.001 (0.233) |
| Sector: Logistics and transport | 0.012 (0.080) | 0.065 (0.218) |
| Sector: Management | 0.013 (0.072) | 0.041 (0.195) |
| Sector: Marketing | -0.101 (0.107) | -0.213 (0.314) |
| Sector: Maintenance | -0.252 (0.204) | -0.946 (0.648) |
| Sector: Education | -0.033 (0.076) | -0.079 (0.192) |
| Sector: Research and development | -0.010 (0.080) | -0.056 (0.220) |
| Sector: Government | -0.059 (0.106) | -0.160 (0.296) |
| Sector: Production | 0.092 (0.111) | 0.192 (0.282) |
| Sector: Technology | 0.193** (0.098) | 0.540** (0.239) |
| Sector: Sales | -0.008 (0.089) | -0.022 (0.256) |
| Sector: Other (reference) | 0.066 (0.091) | 0.099 (0.232) |
| Temporarily unemployed | 0.090 (0.079) | 0.217 (0.199) |
| % of work potentially done via telework | 0.125 (0.083) | 0.239 (0.217) |
| Temporarily extended telework | 0.037 (0.070) | 0.098 (0.180) |
| N | 3,821 | 3,821 |
| Notes. The presented statistics are coefficient estimates and standard errors in parentheses based on a regression analysis with heteroscedasticity-robust standard errors. Intercepts and cut-off values are not presented. * (**) ((***)) indicates significance at the 10% (5%) ((1%)) level. The significance levels cannot be given an absolute interpretation due to potential multiple testing problems (false positives). | | |

| **S1 Table I. Regression Analysis: Evolution in Attaching Importance to Employment Relationship** | | |
| --- | --- | --- |
|  | Linear regression analysis | Ordered logistic regression analysis |
| Female | 0.023 (0.030) | 0.053 (0.073) |
| Age | -0.005*** (0.002) | -0.013*** (0.004) |
| Migration background | -0.147 (0.094) | -0.354* (0.207) |
| Tertiary education | -0.054* (0.030) | -0.127* (0.075) |
| Single (reference) |  |  |
| In a relationship but not cohabiting | 0.025 (0.054) | 0.065 (0.135) |
| In a relationship and cohabiting | -0.039 (0.036) | -0.067 (0.087) |
| Number of resident children | 0.010 (0.013) | 0.016 (0.032) |
| Resident parents | -0.063 (0.058) | -0.126 (0.145) |
| Resident family members (other than parents) | 0.016 (0.074) | -0.016 (0.185) |
| Resident others (not family) | 0.047 (0.081) | 0.119 (0.217) |
| Province of Antwerp (reference) |  |  |
| Province of West Flanders | 0.013 (0.040) | 0.033 (0.094) |
| Province of East Flanders | -0.076** (0.033) | -0.176** (0.082) |
| Province of Limburg | 0.056 (0.051) | 0.117 (0.127) |
| Province of Flemish Brabant | -0.053 (0.040) | -0.122 (0.100) |
| Living in the countryside or rural area (reference) |  |  |
| Living in the centre of a village | -0.081** (0.033) | -0.214*** (0.081) |
| Living in the suburbs of a city | -0.049 (0.035) | -0.111 (0.085) |
| Living in the centre of a city | -0.058 (0.041) | -0.174* (0.101) |
| Health before the COVID-19 crisis (scale) | 0.006 (0.029) | 0.008 (0.063) |
| Current health (scale) | -0.084*** (0.027) | -0.203*** (0.059) |
| Never been a COVID-19 patient (definitely or likely) (reference) |  |  |
| Uncertain about having been a COVID-19 patient | 0.002 (0.032) | 0.021 (0.078) |
| COVID-19 patient at the moment (definitely or likely) | 0.045 (0.077) | 0.122 (0.177) |
| COVID-19 patient in the recent past (definitely or likely) | -0.083 (0.072) | -0.101 (0.176) |
| Employed via a temporary contract in the private sector (reference) |  |  |
| Employed via a permanent contract in the private sector | 0.136** (0.064) | 0.297* (0.165) |
| Employed via a regular contract in the public sector | 0.167** (0.082) | 0.398* (0.210) |
| Employed via a permanent appointment in the public sector | 0.127 (0.079) | 0.281 (0.202) |
| Part−time contract | 0.057 (0.037) | 0.124 (0.090) |
| Tenure with current employer (scale) | 0.008 (0.015) | 0.025 (0.037) |
| Tenure in current job (scale) | 0.006 (0.016) | 0.018 (0.039) |
| Satisfied with job (scale) | -0.082*** (0.017) | -0.210*** (0.039) |
| Autonomous in job (scale) | -0.011 (0.013) | -0.022 (0.030) |
| Dependent on others in job (scale) | 0.020* (0.012) | 0.058** (0.029) |
| Interaction outside of the organisation in job (scale) | 0.021** (0.010) | 0.049* (0.025) |
| Feedback from others in job (scale) | -0.031** (0.013) | -0.074** (0.030) |
| Sector: Purchasing | -0.011 (0.042) | -0.016 (0.099) |
| Sector: Administration | 0.002*** (0.001) | 0.003** (0.001) |
| Sector: Construction | -0.038 (0.042) | -0.057 (0.101) |
| Sector: Communication |  |  |
| Sector: Creative | 0.011 (0.135) | -0.022 (0.341) |
| Sector: Provision of services | 0.008 (0.077) | 0.033 (0.188) |
| Sector: Financial | 0.056 (0.092) | 0.151 (0.226) |
| Sector: Health | -0.078 (0.115) | -0.108 (0.286) |
| Sector: Catering and tourism | 0.132 (0.184) | 0.320 (0.387) |
| Sector: Human Resources | 0.032 (0.074) | 0.087 (0.184) |
| Sector: ICT | 0.023 (0.078) | 0.124 (0.201) |
| Sector: Legal | 0.022 (0.082) | 0.077 (0.200) |
| Sector: Agriculture and horticulture | -0.008 (0.092) | 0.012 (0.225) |
| Sector: Logistics and transport | -0.010 (0.085) | -0.024 (0.213) |
| Sector: Management | 0.057 (0.075) | 0.174 (0.190) |
| Sector: Marketing | -0.069 (0.112) | -0.167 (0.301) |
| Sector: Maintenance | 0.236 (0.262) | 0.495 (0.604) |
| Sector: Education | 0.027 (0.076) | 0.119 (0.185) |
| Sector: Research and development | 0.052 (0.087) | 0.116 (0.215) |
| Sector: Government | -0.122 (0.104) | -0.250 (0.281) |
| Sector: Production | 0.092 (0.105) | 0.236 (0.272) |
| Sector: Technology | 0.087 (0.093) | 0.231 (0.232) |
| Sector: Sales | -0.051 (0.095) | -0.100 (0.247) |
| Sector: Other (reference) | 0.055 (0.092) | 0.123 (0.225) |
| Temporarily unemployed | 0.035 (0.081) | 0.081 (0.195) |
| % of work potentially done via telework | 0.159* (0.086) | 0.396* (0.212) |
| Temporarily extended telework | -0.028 (0.072) | -0.076 (0.177) |
| N | 3,821 | 3,821 |
| Notes. The presented statistics are coefficient estimates and standard errors in parentheses based on a regression analysis with heteroscedasticity-robust standard errors. Intercepts and cut-off values are not presented. * (**) ((***)) indicates significance at the 10% (5%) ((1%)) level. The significance levels cannot be given an absolute interpretation due to potential multiple testing problems (false positives). | | |

| **S1 Table J. Regression Analysis: Evolution in Attaching Importance to Distance to the Workplace** | | |
| --- | --- | --- |
|  | Linear regression analysis | Ordered logistic regression analysis |
| Female | 0.026 (0.033) | 0.041 (0.073) |
| Age | 0.007*** (0.002) | 0.018*** (0.004) |
| Migration background | 0.135 (0.102) | 0.329* (0.200) |
| Tertiary education | -0.067** (0.033) | -0.116 (0.074) |
| Single (reference) |  |  |
| In a relationship but not cohabiting | 0.012 (0.063) | 0.057 (0.137) |
| In a relationship and cohabiting | 0.003 (0.040) | 0.019 (0.087) |
| Number of resident children | 0.005 (0.015) | 0.015 (0.032) |
| Resident parents | 0.019 (0.069) | 0.064 (0.147) |
| Resident family members (other than parents) | -0.024 (0.082) | -0.020 (0.186) |
| Resident others (not family) | -0.189** (0.086) | -0.452** (0.229) |
| Province of Antwerp (reference) |  |  |
| Province of West Flanders | -0.068 (0.042) | -0.147 (0.094) |
| Province of East Flanders | -0.022 (0.037) | -0.071 (0.081) |
| Province of Limburg | 0.000 (0.060) | 0.000 (0.128) |
| Province of Flemish Brabant | -0.053 (0.044) | -0.130 (0.099) |
| Living in the countryside or rural area (reference) |  |  |
| Living in the centre of a village | -0.085** (0.036) | -0.195** (0.080) |
| Living in the suburbs of a city | -0.075* (0.038) | -0.176** (0.085) |
| Living in the centre of a city | -0.022 (0.045) | -0.051 (0.100) |
| Health before the COVID-19 crisis (scale) | 0.015 (0.032) | 0.013 (0.063) |
| Current health (scale) | -0.049* (0.029) | -0.091 (0.059) |
| Never been a COVID-19 patient (definitely or likely) (reference) |  |  |
| Uncertain about having been a COVID-19 patient | 0.041 (0.035) | 0.057 (0.078) |
| COVID-19 patient at the moment (definitely or likely) | 0.034 (0.080) | 0.064 (0.174) |
| COVID-19 patient in the recent past (definitely or likely) | 0.068 (0.086) | 0.160 (0.177) |
| Employed via a temporary contract in the private sector (reference) |  |  |
| Employed via a permanent contract in the private sector | 0.049 (0.073) | 0.108 (0.166) |
| Employed via a regular contract in the public sector | 0.097 (0.095) | 0.245 (0.212) |
| Employed via a permanent appointment in the public sector | 0.116 (0.090) | 0.282 (0.204) |
| Part−time contract | 0.068 (0.041) | 0.112 (0.090) |
| Tenure with current employer (scale) | 0.009 (0.017) | 0.021 (0.037) |
| Tenure in current job (scale) | -0.006 (0.018) | -0.011 (0.039) |
| Satisfied with job (scale) | -0.060*** (0.018) | -0.139*** (0.039) |
| Autonomous in job (scale) | -0.021 (0.014) | -0.049 (0.030) |
| Dependent on others in job (scale) | 0.008 (0.014) | 0.019 (0.029) |
| Interaction outside of the organisation in job (scale) | 0.002 (0.012) | -0.001 (0.025) |
| Feedback from others in job (scale) | -0.014 (0.014) | -0.032 (0.030) |
| Sector: Purchasing | 0.093** (0.046) | 0.238** (0.098) |
| Sector: Administration | 0.001 (0.001) | 0.002 (0.001) |
| Sector: Construction | 0.069 (0.046) | 0.160 (0.101) |
| Sector: Communication |  |  |
| Sector: Creative | 0.037 (0.141) | 0.044 (0.334) |
| Sector: Provision of services | 0.007 (0.086) | 0.026 (0.187) |
| Sector: Financial | 0.024 (0.101) | 0.094 (0.223) |
| Sector: Health | -0.060 (0.131) | -0.173 (0.292) |
| Sector: Catering and tourism | 0.039 (0.172) | 0.142 (0.367) |
| Sector: Human Resources | -0.099 (0.083) | -0.209 (0.185) |
| Sector: ICT | 0.037 (0.090) | 0.122 (0.201) |
| Sector: Legal | 0.064 (0.086) | 0.152 (0.196) |
| Sector: Agriculture and horticulture | -0.200** (0.099) | -0.467** (0.230) |
| Sector: Logistics and transport | -0.047 (0.096) | -0.075 (0.213) |
| Sector: Management | -0.034 (0.085) | -0.027 (0.190) |
| Sector: Marketing | -0.131 (0.153) | -0.303 (0.321) |
| Sector: Maintenance | 0.098 (0.198) | 0.311 (0.554) |
| Sector: Education | 0.015 (0.086) | 0.053 (0.185) |
| Sector: Research and development | -0.040 (0.096) | -0.101 (0.216) |
| Sector: Government | -0.109 (0.117) | -0.215 (0.284) |
| Sector: Production | -0.025 (0.135) | -0.032 (0.281) |
| Sector: Technology | 0.089 (0.106) | 0.199 (0.230) |
| Sector: Sales | -0.024 (0.099) | -0.031 (0.243) |
| Sector: Other (reference) | -0.040 (0.101) | -0.096 (0.223) |
| Temporarily unemployed | 0.061 (0.091) | 0.108 (0.196) |
| % of work potentially done via telework | 0.105 (0.097) | 0.208 (0.212) |
| Temporarily extended telework | -0.060 (0.079) | -0.074 (0.176) |
| N | 3,821 | 3,821 |
| Notes. The presented statistics are coefficient estimates and standard errors in parentheses based on a regression analysis with heteroscedasticity-robust standard errors. Intercepts and cut-off values are not presented. * (**) ((***)) indicates significance at the 10% (5%) ((1%)) level. The significance levels cannot be given an absolute interpretation due to potential multiple testing problems (false positives). | | |

| **S1 Table K. Regression Analysis: Evolution in Attaching Importance to Work-Life Balance** | | |
| --- | --- | --- |
|  | Linear regression analysis | Ordered logistic regression analysis |
| Female | 0.011 (0.031) | 0.020 (0.072) |
| Age | -0.003* (0.002) | -0.007* (0.004) |
| Migration background | 0.059 (0.091) | 0.135 (0.199) |
| Tertiary education | -0.108*** (0.032) | -0.243*** (0.074) |
| Single (reference) |  |  |
| In a relationship but not cohabiting | 0.033 (0.059) | 0.077 (0.136) |
| In a relationship and cohabiting | 0.026 (0.038) | 0.066 (0.086) |
| Number of resident children | 0.035** (0.014) | 0.082*** (0.031) |
| Resident parents | -0.124* (0.066) | -0.267* (0.147) |
| Resident family members (other than parents) | 0.022 (0.083) | 0.012 (0.187) |
| Resident others (not family) | -0.136 (0.093) | -0.302 (0.228) |
| Province of Antwerp (reference) |  |  |
| Province of West Flanders | -0.003 (0.040) | -0.015 (0.093) |
| Province of East Flanders | -0.020 (0.034) | -0.041 (0.080) |
| Province of Limburg | 0.012 (0.056) | 0.025 (0.127) |
| Province of Flemish Brabant | -0.010 (0.043) | -0.024 (0.098) |
| Living in the countryside or rural area (reference) |  |  |
| Living in the centre of a village | -0.083** (0.035) | -0.182** (0.079) |
| Living in the suburbs of a city | -0.045 (0.036) | -0.097 (0.083) |
| Living in the centre of a city | -0.158*** (0.043) | -0.372*** (0.100) |
| Health before the COVID-19 crisis (scale) | 0.010 (0.030) | 0.023 (0.062) |
| Current health (scale) | -0.068** (0.027) | -0.156*** (0.058) |
| Never been a COVID-19 patient (definitely or likely) (reference) |  |  |
| Uncertain about having been a COVID-19 patient | 0.076** (0.033) | 0.177** (0.076) |
| COVID-19 patient at the moment (definitely or likely) | 0.106 (0.076) | 0.243 (0.171) |
| COVID-19 patient in the recent past (definitely or likely) | -0.013 (0.079) | -0.003 (0.176) |
| Employed via a temporary contract in the private sector (reference) |  |  |
| Employed via a permanent contract in the private sector | 0.114* (0.067) | 0.247 (0.164) |
| Employed via a regular contract in the public sector | 0.145* (0.088) | 0.303 (0.209) |
| Employed via a permanent appointment in the public sector | 0.075 (0.083) | 0.153 (0.201) |
| Part−time contract | -0.021 (0.039) | -0.056 (0.088) |
| Tenure with current employer (scale) | 0.012 (0.016) | 0.036 (0.037) |
| Tenure in current job (scale) | 0.009 (0.017) | 0.023 (0.038) |
| Satisfied with job (scale) | -0.087*** (0.017) | -0.210*** (0.038) |
| Autonomous in job (scale) | -0.029** (0.013) | -0.066** (0.029) |
| Dependent on others in job (scale) | 0.031** (0.013) | 0.071** (0.029) |
| Interaction outside of the organisation in job (scale) | 0.017 (0.011) | 0.039 (0.025) |
| Feedback from others in job (scale) | -0.031** (0.013) | -0.071** (0.029) |
| Sector: Purchasing | 0.028 (0.043) | 0.072 (0.096) |
| Sector: Administration | 0.002*** (0.001) | 0.004*** (0.001) |
| Sector: Construction | -0.005 (0.044) | -0.004 (0.099) |
| Sector: Communication |  |  |
| Sector: Creative | -0.025 (0.131) | -0.072 (0.329) |
| Sector: Provision of services | 0.063 (0.080) | 0.183 (0.185) |
| Sector: Financial | 0.123 (0.093) | 0.326 (0.219) |
| Sector: Health | -0.011 (0.128) | 0.027 (0.289) |
| Sector: Catering and tourism | 0.361** (0.164) | 0.866** (0.364) |
| Sector: Human Resources | 0.081 (0.078) | 0.220 (0.182) |
| Sector: ICT | 0.142* (0.084) | 0.363* (0.199) |
| Sector: Legal | 0.210*** (0.081) | 0.495** (0.195) |
| Sector: Agriculture and horticulture | 0.058 (0.096) | 0.116 (0.225) |
| Sector: Logistics and transport | 0.054 (0.092) | 0.156 (0.213) |
| Sector: Management | 0.026 (0.078) | 0.088 (0.188) |
| Sector: Marketing | -0.090 (0.124) | -0.191 (0.308) |
| Sector: Maintenance | 0.142 (0.298) | 0.320 (0.626) |
| Sector: Education | 0.033 (0.081) | 0.099 (0.185) |
| Sector: Research and development | 0.078 (0.093) | 0.222 (0.213) |
| Sector: Government | -0.078 (0.108) | -0.159 (0.280) |
| Sector: Production | 0.100 (0.118) | 0.204 (0.275) |
| Sector: Technology | 0.246** (0.099) | 0.600*** (0.230) |
| Sector: Sales | -0.068 (0.094) | -0.095 (0.244) |
| Sector: Other (reference) | 0.122 (0.094) | 0.332 (0.221) |
| Temporarily unemployed | 0.098 (0.082) | 0.272 (0.191) |
| % of work potentially done via telework | 0.102 (0.089) | 0.249 (0.210) |
| Temporarily extended telework | 0.026 (0.074) | 0.099 (0.174) |
| N | 3,821 | 3,821 |
| Notes. The presented statistics are coefficient estimates and standard errors in parentheses based on a regression analysis with heteroscedasticity-robust standard errors. Intercepts and cut-off values are not presented. * (**) ((***)) indicates significance at the 10% (5%) ((1%)) level. The significance levels cannot be given an absolute interpretation due to potential multiple testing problems (false positives). | | |

| **S1 Table L. Regression Analysis: Evolution in Attaching Importance to Working Conditions** | | |
| --- | --- | --- |
|  | Linear regression analysis | Ordered logistic regression analysis |
| Female | 0.031 (0.030) | 0.064 (0.072) |
| Age | -0.001 (0.002) | -0.002 (0.004) |
| Migration background | 0.000 (0.084) | 0.025 (0.197) |
| Tertiary education | -0.064** (0.030) | -0.170** (0.074) |
| Single (reference) |  |  |
| In a relationship but not cohabiting | -0.036 (0.055) | -0.092 (0.134) |
| In a relationship and cohabiting | -0.049 (0.035) | -0.086 (0.085) |
| Number of resident children | 0.002 (0.013) | 0.001 (0.032) |
| Resident parents | 0.072 (0.059) | 0.245* (0.143) |
| Resident family members (other than parents) | -0.050 (0.076) | -0.161 (0.184) |
| Resident others (not family) | 0.003 (0.088) | 0.060 (0.217) |
| Province of Antwerp (reference) |  |  |
| Province of West Flanders | 0.024 (0.038) | 0.054 (0.093) |
| Province of East Flanders | 0.017 (0.033) | 0.051 (0.081) |
| Province of Limburg | 0.065 (0.051) | 0.137 (0.126) |
| Province of Flemish Brabant | -0.020 (0.039) | -0.045 (0.099) |
| Living in the countryside or rural area (reference) |  |  |
| Living in the centre of a village | -0.062* (0.032) | -0.151* (0.079) |
| Living in the suburbs of a city | -0.013 (0.034) | -0.010 (0.084) |
| Living in the centre of a city | -0.082** (0.041) | -0.195* (0.100) |
| Health before the COVID-19 crisis (scale) | 0.038 (0.028) | 0.095 (0.063) |
| Current health (scale) | -0.096*** (0.026) | -0.240*** (0.059) |
| Never been a COVID-19 patient (definitely or likely) (reference) |  |  |
| Uncertain about having been a COVID-19 patient | 0.028 (0.031) | 0.057 (0.076) |
| COVID-19 patient at the moment (definitely or likely) | 0.083 (0.073) | 0.221 (0.173) |
| COVID-19 patient in the recent past (definitely or likely) | -0.033 (0.071) | -0.050 (0.175) |
| Employed via a temporary contract in the private sector (reference) |  |  |
| Employed via a permanent contract in the private sector | 0.084 (0.066) | 0.194 (0.163) |
| Employed via a regular contract in the public sector | 0.105 (0.084) | 0.226 (0.209) |
| Employed via a permanent appointment in the public sector | 0.062 (0.079) | 0.167 (0.199) |
| Part−time contract | 0.006 (0.036) | 0.024 (0.089) |
| Tenure with current employer (scale) | 0.006 (0.015) | 0.023 (0.037) |
| Tenure in current job (scale) | -0.001 (0.016) | -0.001 (0.039) |
| Satisfied with job (scale) | -0.086*** (0.016) | -0.233*** (0.038) |
| Autonomous in job (scale) | -0.018 (0.012) | -0.039 (0.030) |
| Dependent on others in job (scale) | 0.019 (0.012) | 0.050* (0.029) |
| Interaction outside of the organisation in job (scale) | 0.016 (0.010) | 0.040 (0.025) |
| Feedback from others in job (scale) | -0.040*** (0.013) | -0.098*** (0.030) |
| Sector: Purchasing | -0.038 (0.041) | -0.079 (0.097) |
| Sector: Administration | 0.001** (0.001) | 0.002* (0.001) |
| Sector: Construction | -0.056 (0.040) | -0.130 (0.099) |
| Sector: Communication |  |  |
| Sector: Creative | 0.012 (0.124) | 0.007 (0.329) |
| Sector: Provision of services | -0.003 (0.077) | 0.037 (0.188) |
| Sector: Financial | 0.088 (0.090) | 0.303 (0.221) |
| Sector: Health | -0.054 (0.123) | -0.004 (0.290) |
| Sector: Catering and tourism | 0.254* (0.148) | 0.632* (0.365) |
| Sector: Human Resources | 0.105 (0.076) | 0.313* (0.183) |
| Sector: ICT | -0.008 (0.080) | 0.035 (0.202) |
| Sector: Legal | 0.187** (0.080) | 0.526*** (0.196) |
| Sector: Agriculture and horticulture | 0.035 (0.092) | 0.115 (0.224) |
| Sector: Logistics and transport | -0.039 (0.081) | -0.016 (0.210) |
| Sector: Management | 0.051 (0.076) | 0.172 (0.190) |
| Sector: Marketing | -0.221* (0.113) | -0.514* (0.305) |
| Sector: Maintenance | 0.186 (0.294) | 0.378 (0.639) |
| Sector: Education | -0.011 (0.077) | 0.064 (0.185) |
| Sector: Research and development | 0.041 (0.087) | 0.141 (0.214) |
| Sector: Government | -0.076 (0.107) | -0.158 (0.282) |
| Sector: Production | 0.152 (0.115) | 0.413 (0.272) |
| Sector: Technology | 0.094 (0.096) | 0.326 (0.232) |
| Sector: Sales | -0.106 (0.093) | -0.246 (0.249) |
| Sector: Other (reference) | 0.088 (0.092) | 0.271 (0.224) |
| Temporarily unemployed | 0.092 (0.082) | 0.289 (0.194) |
| % of work potentially done via telework | 0.157* (0.083) | 0.375* (0.210) |
| Temporarily extended telework | 0.027 (0.073) | 0.096 (0.176) |
| N | 3,821 | 3,821 |
| Notes. The presented statistics are coefficient estimates and standard errors in parentheses based on a regression analysis with heteroscedasticity-robust standard errors. Intercepts and cut-off values are not presented. * (**) ((***)) indicates significance at the 10% (5%) ((1%)) level. The significance levels cannot be given an absolute interpretation due to potential multiple testing problems (false positives). | | |

| **S1 Table M. Regression Analysis: Evolution in Attaching Importance to Wage** | | |
| --- | --- | --- |
|  | Linear regression analysis | Ordered logistic regression analysis |
| Female | -0.019 (0.026) | -0.020 (0.082) |
| Age | -0.008*** (0.001) | -0.026*** (0.004) |
| Migration background | -0.013 (0.086) | -0.110 (0.225) |
| Tertiary education | -0.120*** (0.027) | -0.386*** (0.084) |
| Single (reference) |  |  |
| In a relationship but not cohabiting | 0.054 (0.050) | 0.166 (0.147) |
| In a relationship and cohabiting | -0.080** (0.032) | -0.259*** (0.097) |
| Number of resident children | 0.008 (0.012) | 0.024 (0.036) |
| Resident parents | -0.017 (0.056) | -0.050 (0.159) |
| Resident family members (other than parents) | -0.016 (0.070) | -0.130 (0.205) |
| Resident others (not family) | -0.068 (0.084) | -0.272 (0.253) |
| Province of Antwerp (reference) |  |  |
| Province of West Flanders | 0.021 (0.034) | 0.084 (0.105) |
| Province of East Flanders | 0.008 (0.029) | 0.029 (0.091) |
| Province of Limburg | 0.024 (0.047) | 0.080 (0.143) |
| Province of Flemish Brabant | -0.012 (0.036) | -0.058 (0.113) |
| Living in the countryside or rural area (reference) |  |  |
| Living in the centre of a village | -0.013 (0.029) | -0.031 (0.090) |
| Living in the suburbs of a city | 0.037 (0.031) | 0.136 (0.095) |
| Living in the centre of a city | -0.017 (0.037) | 0.008 (0.114) |
| Health before the COVID-19 crisis (scale) | 0.001 (0.028) | 0.015 (0.071) |
| Current health (scale) | -0.036 (0.025) | -0.136** (0.066) |
| Never been a COVID-19 patient (definitely or likely) (reference) |  |  |
| Uncertain about having been a COVID-19 patient | 0.046 (0.028) | 0.106 (0.087) |
| COVID-19 patient at the moment (definitely or likely) | 0.013 (0.065) | 0.013 (0.195) |
| COVID-19 patient in the recent past (definitely or likely) | -0.001 (0.065) | -0.057 (0.198) |
| Employed via a temporary contract in the private sector (reference) |  |  |
| Employed via a permanent contract in the private sector | 0.021 (0.057) | 0.144 (0.182) |
| Employed via a regular contract in the public sector | 0.069 (0.075) | 0.310 (0.237) |
| Employed via a permanent appointment in the public sector | 0.011 (0.069) | 0.141 (0.225) |
| Part−time contract | -0.010 (0.031) | -0.027 (0.101) |
| Tenure with current employer (scale) | -0.020 (0.013) | -0.067 (0.042) |
| Tenure in current job (scale) | 0.014 (0.014) | 0.044 (0.044) |
| Satisfied with job (scale) | -0.005 (0.015) | -0.012 (0.043) |
| Autonomous in job (scale) | 0.017 (0.012) | 0.066** (0.034) |
| Dependent on others in job (scale) | -0.010 (0.011) | -0.038 (0.033) |
| Interaction outside of the organisation in job (scale) | 0.019** (0.009) | 0.073*** (0.028) |
| Feedback from others in job (scale) | -0.013 (0.011) | -0.033 (0.033) |
| Sector: Purchasing | -0.000 (0.039) | 0.035 (0.109) |
| Sector: Administration | 0.001* (0.000) | 0.003** (0.001) |
| Sector: Construction | -0.161*** (0.036) | -0.524*** (0.113) |
| Sector: Communication |  |  |
| Sector: Creative | -0.026 (0.116) | -0.035 (0.374) |
| Sector: Provision of services | -0.066 (0.067) | -0.190 (0.208) |
| Sector: Financial | -0.163* (0.089) | -0.509** (0.257) |
| Sector: Health | -0.007 (0.099) | 0.131 (0.321) |
| Sector: Catering and tourism | -0.243* (0.130) | -0.790* (0.426) |
| Sector: Human Resources | -0.106 (0.066) | -0.221 (0.204) |
| Sector: ICT | -0.068 (0.067) | -0.090 (0.227) |
| Sector: Legal | 0.010 (0.072) | 0.133 (0.220) |
| Sector: Agriculture and horticulture | 0.027 (0.086) | 0.094 (0.245) |
| Sector: Logistics and transport | -0.041 (0.074) | -0.097 (0.235) |
| Sector: Management | 0.002 (0.068) | 0.075 (0.213) |
| Sector: Marketing | 0.022 (0.105) | 0.192 (0.329) |
| Sector: Maintenance | -0.040 (0.191) | 0.130 (0.622) |
| Sector: Education | -0.083 (0.070) | -0.175 (0.207) |
| Sector: Research and development | -0.077 (0.075) | -0.130 (0.239) |
| Sector: Government | -0.107 (0.087) | -0.261 (0.316) |
| Sector: Production | 0.114 (0.110) | 0.413 (0.297) |
| Sector: Technology | -0.038 (0.083) | -0.071 (0.263) |
| Sector: Sales | -0.177** (0.076) | -0.477* (0.277) |
| Sector: Other (reference) | -0.001 (0.082) | 0.073 (0.251) |
| Temporarily unemployed | -0.054 (0.073) | -0.096 (0.217) |
| % of work potentially done via telework | 0.074 (0.081) | 0.258 (0.235) |
| Temporarily extended telework | -0.057 (0.065) | -0.146 (0.195) |
| N | 3,821 | 3,821 |
| Notes. The presented statistics are coefficient estimates and standard errors in parentheses based on a regression analysis with heteroscedasticity-robust standard errors. Intercepts and cut-off values are not presented. * (**) ((***)) indicates significance at the 10% (5%) ((1%)) level. The significance levels cannot be given an absolute interpretation due to potential multiple testing problems (false positives). | | |

| **S1 Table N. Regression Analysis: Evolution in Attaching Importance to Possibility of Teleworking** | | |
| --- | --- | --- |
|  | Linear regression analysis | Ordered logistic regression analysis |
| Female | 0.101*** (0.036) | 0.179** (0.070) |
| Age | -0.005** (0.002) | -0.008** (0.004) |
| Migration background | 0.009 (0.108) | 0.041 (0.193) |
| Tertiary education | -0.009 (0.037) | -0.034 (0.072) |
| Single (reference) |  |  |
| In a relationship but not cohabiting | 0.130* (0.068) | 0.238* (0.131) |
| In a relationship and cohabiting | 0.010 (0.045) | 0.006 (0.084) |
| Number of resident children | -0.001 (0.016) | -0.001 (0.031) |
| Resident parents | -0.088 (0.077) | -0.104 (0.140) |
| Resident family members (other than parents) | -0.121 (0.093) | -0.309* (0.178) |
| Resident others (not family) | -0.009 (0.118) | 0.039 (0.220) |
| Province of Antwerp (reference) |  |  |
| Province of West Flanders | 0.007 (0.048) | 0.025 (0.091) |
| Province of East Flanders | 0.026 (0.042) | 0.076 (0.079) |
| Province of Limburg | 0.049 (0.064) | 0.090 (0.123) |
| Province of Flemish Brabant | 0.034 (0.049) | 0.056 (0.096) |
| Living in the countryside or rural area (reference) |  |  |
| Living in the centre of a village | -0.038 (0.040) | -0.074 (0.077) |
| Living in the suburbs of a city | -0.067 (0.043) | -0.126 (0.082) |
| Living in the centre of a city | -0.103** (0.050) | -0.204** (0.097) |
| Health before the COVID-19 crisis (scale) | 0.085** (0.033) | 0.151** (0.061) |
| Current health (scale) | -0.075** (0.031) | -0.142** (0.057) |
| Never been a COVID-19 patient (definitely or likely) (reference) |  |  |
| Uncertain about having been a COVID-19 patient | 0.051 (0.040) | 0.104 (0.075) |
| COVID-19 patient at the moment (definitely or likely) | 0.066 (0.093) | 0.132 (0.172) |
| COVID-19 patient in the recent past (definitely or likely) | 0.063 (0.089) | 0.125 (0.170) |
| Employed via a temporary contract in the private sector (reference) |  |  |
| Employed via a permanent contract in the private sector | 0.112 (0.090) | 0.170 (0.160) |
| Employed via a regular contract in the public sector | 0.300*** (0.111) | 0.549*** (0.205) |
| Employed via a permanent appointment in the public sector | 0.172 (0.105) | 0.278 (0.195) |
| Part−time contract | 0.035 (0.047) | 0.066 (0.088) |
| Tenure with current employer (scale) | 0.018 (0.018) | 0.028 (0.036) |
| Tenure in current job (scale) | -0.012 (0.019) | -0.007 (0.038) |
| Satisfied with job (scale) | -0.063*** (0.020) | -0.119*** (0.037) |
| Autonomous in job (scale) | 0.008 (0.016) | 0.021 (0.029) |
| Dependent on others in job (scale) | 0.014 (0.015) | 0.024 (0.028) |
| Interaction outside of the organisation in job (scale) | 0.005 (0.013) | 0.004 (0.024) |
| Feedback from others in job (scale) | -0.040** (0.015) | -0.085*** (0.029) |
| Sector: Purchasing | 0.190*** (0.054) | 0.358*** (0.096) |
| Sector: Administration | 0.008*** (0.001) | 0.015*** (0.001) |
| Sector: Construction | 0.200*** (0.051) | 0.350*** (0.097) |
| Sector: Communication |  |  |
| Sector: Creative | 0.117 (0.157) | 0.172 (0.320) |
| Sector: Provision of services | 0.240*** (0.093) | 0.507*** (0.179) |
| Sector: Financial | 0.079 (0.118) | 0.210 (0.216) |
| Sector: Health | 0.163 (0.133) | 0.375 (0.271) |
| Sector: Catering and tourism | 0.273 (0.203) | 0.476 (0.366) |
| Sector: Human Resources | 0.075 (0.092) | 0.131 (0.175) |
| Sector: ICT | 0.252*** (0.096) | 0.532*** (0.194) |
| Sector: Legal | -0.036 (0.102) | 0.012 (0.190) |
| Sector: Agriculture and horticulture | -0.070 (0.115) | -0.068 (0.215) |
| Sector: Logistics and transport | 0.178* (0.103) | 0.344* (0.202) |
| Sector: Management | 0.174* (0.092) | 0.345* (0.182) |
| Sector: Marketing | -0.037 (0.149) | -0.046 (0.286) |
| Sector: Maintenance | 0.038 (0.207) | 0.045 (0.533) |
| Sector: Education | 0.025 (0.094) | 0.084 (0.177) |
| Sector: Research and development | 0.278*** (0.099) | 0.534*** (0.203) |
| Sector: Government | 0.167 (0.120) | 0.302 (0.263) |
| Sector: Production | -0.239* (0.141) | -0.494* (0.261) |
| Sector: Technology | 0.079 (0.116) | 0.147 (0.223) |
| Sector: Sales | 0.018 (0.110) | 0.027 (0.232) |
| Sector: Other (reference) | 0.058 (0.112) | 0.176 (0.215) |
| Temporarily unemployed | -0.020 (0.100) | -0.024 (0.185) |
| % of work potentially done via telework | 0.207* (0.112) | 0.434** (0.206) |
| Temporarily extended telework | 0.167* (0.088) | 0.340** (0.168) |
| N | 3,821 | 3,821 |
| Notes. The presented statistics are coefficient estimates and standard errors in parentheses based on a regression analysis with heteroscedasticity-robust standard errors. Intercepts and cut-off values are not presented. * (**) ((***)) indicates significance at the 10% (5%) ((1%)) level. The significance levels cannot be given an absolute interpretation due to potential multiple testing problems (false positives). | | |

| **S1 Table O. Wage-earning inhabitants of Flanders (15-64 years) by sex and level of education** | | | | |
| --- | --- | --- | --- | --- |
| Sex | Age | Level of education | Absolute number | Proportion in % |
| Male | 15-24 years | Low | 27,443 | 1.1 |
|  |  | Average | 66,996 | 2.6 |
|  |  | High | 24,998 | 1.0 |
|  | 25-49 years | Low | 109,779 | 4.3 |
|  |  | Average | 358,070 | 14.1 |
|  |  | High | 322,429 | 12.7 |
|  | 50-64 years | Low | 83,380 | 3.3 |
|  |  | Average | 166,225 | 6.6 |
|  |  | High | 138,000 | 5.4 |
| Female | 15-24 years | Low | 17,620 | 0.7 |
|  |  | Average | 52,745 | 2.1 |
|  |  | High | 37,350 | 1.5 |
|  | 25-49 years | Low | 57,542 | 2.3 |
|  |  | Average | 276,715 | 10.6 |
|  |  | High | 450,674 | 17.8 |
|  | 50-64 years | Low | 55,613 | 2.2 |
|  |  | Average | 154,056 | 6.1 |
|  |  | High | 144,496 | 5.7 |
| Total |  |  | 2,535,131 | 100 |
| Notes. The population statistics for 2019 were provided by the General Directorate of Statistics—Statistics Belgium. Inhabitants with a low level of education have at maximum completed lower secondary education. Inhabitants with an average level of education acquired a degree of higher secondary education. Inhabitants have a high level of education when they completed higher education. | | | | |

| **SS1 Table P. Correlation matrix between the temporarily unemployed and the three variables used for post-stratification (Gender, Age and Level of education)** | | | | |
| --- | --- | --- | --- | --- |
|  | Temporarily unemployed | Gender (female) | Age | Level of education (tertiary) |
| Temporarily unemployed | 1.00 | .05 | -.03 | -.20 |
| Gender (female) | .05 | 1.00 | -.07 | .14 |
| Age | -.03 | -.07 | 1.00 | -.15 |
| Level of education (tertiary) | -.20 | .14 | -.15 | 1.00 |
|  | | | | |
